# Diffusiophoretic transport of colloids in porous media

**DOI:** 10.1126/sciadv.ady9874

**Published:** 2026-02-11

**Authors:** Mobin Alipour, Yiran Li, Haoyu Liu, Amir A. Pahlavan

**Affiliations:** Department of Mechanical Engineering and Materials Science, Yale University, New Haven, CT 06511, USA.

## Abstract

Chemical gradients are ubiquitous in porous media flows, from tidal salt gradients in aquifers to irrigation-driven gradients in soils and ionic gradients from metabolic activity in tissues. Although chemical gradients are known to drive diffusiophoretic migration of colloids, these nonequilibrium forces have largely been ignored in porous media flows. Under typical subsurface conditions, flow velocities within preferential pathways exceed phoretic velocities by orders of magnitude, suggesting that diffusiophoresis would be limited to stagnant pockets. Here, using microfluidic experiments, numerical simulations, and theoretical modeling, we show that even moderate solute gradients, typical of natural mixing, can markedly alter colloid transport. We uncover a previously overlooked effect: cross-streamline phoretic migration within preferential flow pathways, which changes macroscopic dispersion by orders of magnitude and suppresses the impact of geometric disorder on transport. Our findings challenge classical models of colloid transport, highlighting the broad implications of solute gradients for technological, biomedical, and environmental applications.

## Teaser

Solute gradients drive cross-streamline migration of colloids in flow pathways, substantially affecting macroscopic transport.

## INTRODUCTION

Chemical gradients lead to the migration of colloids in a process known as diffusiophoresis (*1*–*6*). These gradients are ubiquitous in porous media flows with potential to affect a myriad of transport processes (*7*–*21*). In agricultural runoff zones, fertilizer and pesticide gradients could modulate the retention and migration of microplastics in soils, influencing their long-term accumulation and impact on soil structure and microbial populations (*22*–*28*). In subsurface remediation efforts, chemical gradients due to dissolved pollutants or redox reactions could be used to guide the nanoparticles such as nanoscale zero-valent iron (nZVI) toward contaminant hotspots, enhancing their retention and efficiency (*29*–*32*). In Arctic regions, permafrost thaw and freeze cycles create strong salinity gradients, potentially influencing the migration of natural and anthropogenic colloids into Arctic rivers and lakes, affecting the biogeochemical cycles (*33*–*39*). Natural and synthetic colloids can also act as contaminant carriers, adsorbing and spreading pesticides, heavy metals, and pathogens over large distances, threatening water resources and ecological biodiversity (*40*–*46*). Therefore, predicting and controlling the transport of colloids in porous media flows is essential.

The nonequilibrium effects of chemical gradients on colloid transport in porous media, however, have been largely ignored thus far (*47*–*70*). In typical subsurface flow scenarios, background flow velocities within preferential flow pathways are orders of magnitude stronger (*U* ≈ 100 μm/s) than the expected phoretic velocities ($u_{\text{dp}}=\Gamma_{p}\nabla \text{ln}c\approx 1\;\mu m/s$), where $\Gamma_{p}$ is the diffusiophoretic mobility and *c* is the solute concentration. Consequently, one might expect diffusiophoresis to be limited to stagnant pockets or dead-end pores. Recent studies support this view, demonstrating the effectiveness of diffusiophoresis in transporting colloids in porous gels and biofilms in the absence of flows (*71*–*75*) or within stagnant fluid pockets even when background flows are present (*76*–*79*).

Here, we present an experimental investigation of diffusiophoretic transport of colloids in porous media flows, uncovering a previously overlooked but substantial effect: the cross-streamline migration of colloids within convective pores, which can alter transport times and macroscopic dispersion by orders of magnitude. By systematically varying the geometric disorder of the medium, we probe how the interplay between flow heterogeneity and diffusiophoresis shapes colloid transport. Contrary to the expectations that diffusiophoresis primarily influences dead-end pores or stagnant zones, our results demonstrate its pervasive impact within flow pathways, highlighting the need to revisit classical models of colloid dispersion in porous flows.

## RESULTS

**Fig. 1.**
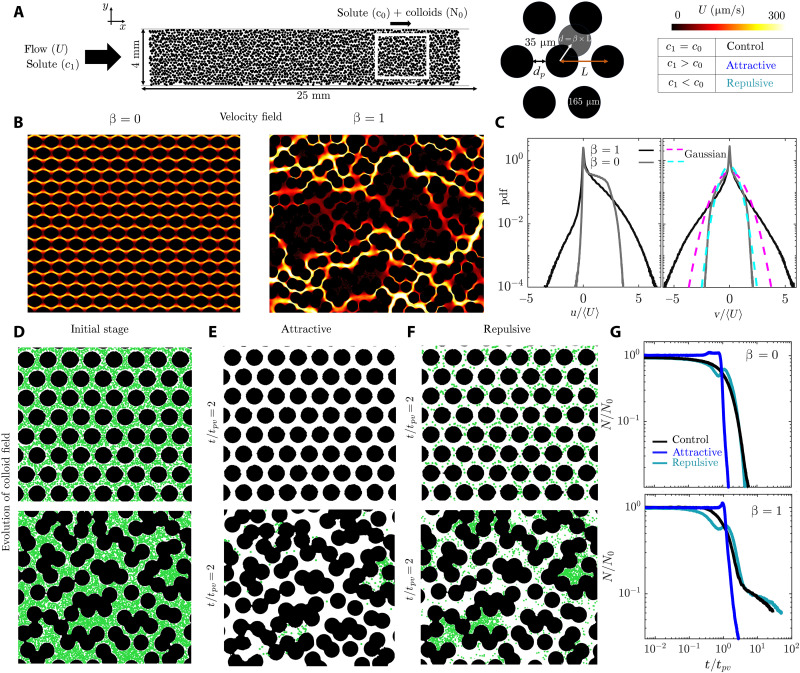
Solute gradients modulate the macroscopic transport of colloids in both ordered and disordered media. (**A**) Schematic of the microfluidic chips patterned with obstacles. The field of view (FOV) of the experiments is marked with the white rectangle. We introduce geometric disorder into an ordered lattice by randomly displacing the obstacles with an amplitude βL, where the perturbation amplitude 0 < β < 1, and *L* represents the center to center spacing between the obstacles in the ordered lattice. (**B**) Whereas flow in the ordered lattice (β = 0) is periodic, geometric disorder (β = 1) leads to the emergence of preferential flow pathways surrounding stagnant fluid pockets/dead-end pores. (**C**) The geometric disorder therefore broadens the probability distribution of both the longitudinal and transverse components of the velocity field and leads to the deviation of the transverse component from the Gaussian distribution. (**D**) Displacing the initially uniformly dispersed colloids (green dots) in both ordered and disordered media with a front with higher (attractive)/lower (repulsive) solute concentration (**E** and **F**), we then monitor how colloids get removed from the medium. (**G**) The time evolution of normalized number of colloids, $N\left(t\right)/N_{0}$, clearly demonstrates the influence of solute gradients on the macroscopic transport of colloids. Here, we have *c*_1_/*c*_0_ ≈ 100 in the attractive case and *c*_1_/*c*_0_ ≈ 0.01 in the repulsive case. [(B) to (G)] Experimental data (see Materials and Methods for details of particle tracking).

We fabricate microfluidic chips patterned with an ordered array of obstacles and introduce disorder by randomly perturbing their position. The amplitude of the perturbations influences both the flow velocity distribution (Fig. 1A) (*80*, *81*), and fraction of dead-end pores in the medium (sections S1 and S2). We fabricate chips with six different perturbation amplitudes (β = 0, 0.2, 0.4, 0.6, 0.8, and 1), noting that β = 0, 0.2 designs do not include any dead-end pores (fig. S3). Flow in the ordered arrays (β = 0) is periodic, whereas disorder (β > 0) leads to the emergence of high-velocity preferential flow pathways that surround the low-velocity pockets in the medium (Fig. 1B). Geometric disorder broadens the velocity field distribution in the medium and leads to its deviation from the Gaussian distribution (Fig. 1C and sections S1 and S2).

We fill the medium with a dilute aqueous solution of negatively charged 1-μm-diameter colloids (green dots in Fig. 1D) with a background solute concentration *c*_0_ (LiCl; see section S3 for the influence of salt type) and then flush the colloids out with a second aqueous solution with a solute concentration *c*_1_ (Fig. 1, E and F). This displacement scenario is representative of many subsurface processes, from irrigation and pesticide discharge in agriculture to contaminant remediation and permafrost thawing, where flows and solute gradients could mobilize natural or anthropogenic colloids, and their evolution needs to be predicted and monitored (*8*). We denote the case with *c*_1_ = *c*_0_ as our “control” case, where solute gradients are absent. When *c*_1_ > *c*_0_, the colloids will be “attracted” to the invading solute front, whereas for *c*_1_ < *c*_0_, they will be “repelled” by the solute front; we therefore denote these two cases as “attractive” and “repulsive,” respectively. We then monitor the evolution of the colloidal density in a field of view downstream in the medium (Fig. 1A). The panels in Fig. 1 (E and F) show snapshots of the evolving colloidal density field (green dots) in both ordered and disordered media. The top row corresponds to the ordered medium, displaying the initial colloid distribution before the solute front arrives, and the same field of view after flushing by the solute front at *t*/*t*_pv_ = 2 for the attractive case with *c*_1_/*c*_0_ = 100 (middle column) and the repulsive case with *c*_1_/*c*_0_ = 0.01 (right column). The bottom row presents the corresponding snapshots for the disordered medium with β = 1. The pore volume timescale $t_{\text{pv}}$ is defined as the time required to displace the entire chip volume at the mean velocity of the medium, $t_{\text{pv}}=V/Q$, where V=LWhϕ with L,W,H,ϕ, and *Q* representing the medium length, width, height, porosity, and flow rate, respectively.

### Solute gradients modulate the macroscopic transport of colloids in both ordered and disordered media

The transport of colloids in the absence of solute gradients, i.e., the control case, is modulated by the flow velocity distribution in the medium. In the ordered lattices, the colloid density decreases almost exponentially in time and is characterized as “Fickian” (black line in Fig. 1G and section S4). In the presence of disorder, this regime is followed by a non-Fickian power-law regime with $N/N_{0}∼t^{-m}$, where, generally, 0 < *m* < 2 with *N*(*t*) representing the total number of colloids in the field of view and $N_{0}=N\left(t=0\right)$. This transition from Fickian to non-Fickian behavior is due to geometric disorder and its effect on the velocity distribution in the medium (*82*–*90*). In stagnant pockets of the medium, diffusion is the dominant escape route for the colloids, leading to their long residence time and the emergence of the power-law behavior.

Adding a “pinch of salt,” however, drastically changes this picture. In both ordered and disordered media, the attractive solute front displaces the colloids much more effectively than the control case (Fig. 1, D and E), shortening their macroscopic transport timescale through the medium as the time evolution of colloid density demonstrates (blue versus black curves in Fig. 1G). This rapid removal of colloids suppresses the non-Fickian regime of transport in the disordered case (β = 1), evidenced by the absence of the power-law tail. In contrast, the repulsive solute front leads to weak changes in the evolution of colloid density field compared to the control case (Fig. 1, F and G). Although the mean flow velocity in a porous medium can exceed typical diffusiophoretic velocities by orders of magnitude, solute gradients can still drive colloid migration in stagnant fluid pockets or dead-end pores where flow is negligible (*77*–*79*). This raises a key question: Could this mechanism, i.e., the diffusiophoretic exchange of particles between the dead-end pores and main flow pathways, underlie our observations?

### Solute gradients modulate the fraction of colloids in the dead-end pores

**Fig. 2.**
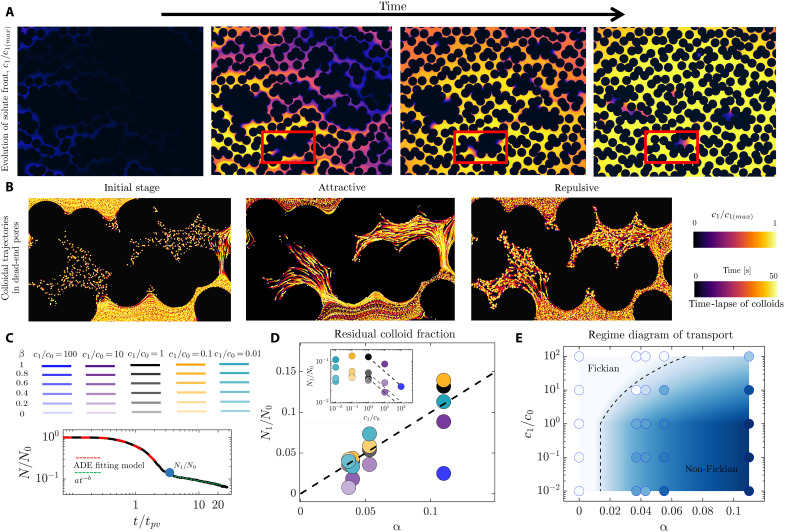
Solute gradients modulate the fraction of colloids in the dead-end pores and the transition between Fickian and non-Fickian regimes of transport. (**A** and **B**) Using dual-channel imaging, we monitor the evolution of both the solute front tagged with a dilute concentration of fluorescein and the fluorescent colloids. We note that the difference in diffusivity of fluorescein and LiCl makes this technique an approximate probe for the LiCl front. The solute gradients around the dead-end pores drive the phoretic migration of colloids out of these regions in the attractive case and into them in the repulsive case, consistent with earlier experiments and simulations (*77*–*79*). (**C**) The residual density of colloids at the onset of non-Fickian regime ($N_{1}/N_{0}$) is proportional to the area fraction of dead-end pores $N_{1}/N_{0}∼\alpha$ (**D**). It is, however, also modulated by the solute gradients (inset). Whereas the repulsive front weakly influences the residual colloid density, the attractive front removes the colloids from the dead-end pores, suppressing the non-Fickian regime. The dashed line in the inset represents $N_{1}/N_{0}\approx \alpha {\left(c_{1}/c_{0}\right)}^{-\Gamma_{p}/D_{s}}$ (section S6). (**E**) Regime diagram of the macroscopic transport as characterized by the residual colloid density $N_{1}/N_{0}$, where circles represent the experimental data and the background color map represents our model. The dashed line is a representative contour line corresponding to $N_{1}/N_{0}=0.01$ as a threshold, separating Fickian and non-Fickian regimes.

Colloidal diffusion alone is too slow to transport particles across these regions; for example, a 1-μm-colloid with diffusivity $D_{p}\approx 10^{-13}\;m^{2}/s$ requires $L^{2}/D_{p}\approx 10^{5}\;s$ to traverse *L* ≈ 100 μm. In contrast, a molecular solute with diffusivity $D_{s}\approx 10^{-9}\;m^{2}/s$ covers the same distance roughly four orders of magnitude faster ($L^{2}/D_{s}\approx 10\;s$), driving the diffusiophoretic migration of colloids into and out of dead-end pores (*91*–*101*). We observe that the phoretic migration of colloids into/out of dead-end pores in the porous medium persists almost an order of magnitude longer, i.e., O(100 s) (Fig. 2, A and B). This persistence is due to the dispersion of the solute front in the porous medium, leading to a gradual rather than a sharp change in solute concentration around stagnant pockets (section S5) (*102*–*106*). The solute and particle Péclet number in our experiments are ${\text{Pe}}_{s}={\mathrm{Ud}}_{p}/D_{s}\approx 10$ and ${\text{Pe}}_{p}={\mathrm{Ud}}_{p}/D_{s}\approx 10^{5}$, where $d_{p}$ is the pore size and *U* is the mean flow velocity.

Phoretic migration modulates the fraction of colloids in dead-end pores, as quantified by the ratio $N_{1}/N_{0}$ at the transition between early-time Fickian and late-time non-Fickian transport regimes (Fig. 2C). This ratio depends on the fraction of dead-end pores α and the presence of solute gradients (Fig. 2D). For an attractive solute front, the residual particle fraction can be expressed as $N_{1}/N_{0}\approx \alpha {\left(c_{1}/c_{0}\right)}^{-\Gamma_{p}/D_{s}}$ (Fig. 2D, inset, and section S6). We note that the work of (*78*) also developed a model for residual particle fraction, assuming a sharp solute front. When the $N_{1}/N_{0}$ ratio goes below 0.01, we classify the transport as Fickian (dashed line in the regime diagram of Fig. 2E). These observations demonstrate that solute gradients can shift the macroscopic transport regime from non-Fickian to Fickian, in agreement with previous experiments and simulations showing the role of diffusiophoresis in modulating colloid/emulsion fraction in dead-end pores (*77*–*79*).

We also observe a pronounced effect of solute gradients on colloid travel times even in ordered lattices without dead-end pores (Fig. 1G). This raises a key question: Is the influence of diffusiophoresis restricted to dead-end pores? Addressing this question requires probing colloidal dynamics at the pore scale.

### Solute gradients modulate the trajectories and velocity statistics of colloids

**Fig. 3.**
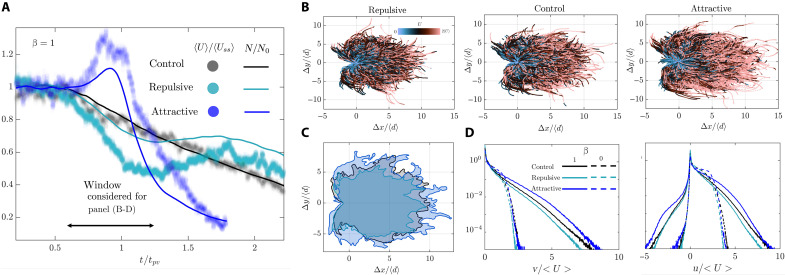
Solute gradients modulate the trajectories and velocity statistics of colloids. (**A**) The temporal evolution of the normalized mean colloid velocity (symbols) and density fields (lines) corresponding to the disordered medium (β = 1). In the control case, we observe a monotonic decrease in the colloid density field, leading to a proportional decrease in the mean velocity of colloids as the faster colloids travel through the domain, leaving the slower ones behind. The attractive and repulsive cases, however, strongly deviate from this trend, showing a net transient increase in the mean colloid density and velocity in the attractive case and an opposite effect in the repulsive case. (**B**) Following the trajectories of the colloids, we observe the signature of the solute gradients in suppressing the transverse dispersion in the repulsive case while enhancing it in the attractive case. The displacement of colloids is normalized by the mean pore size 〈d〉. (**C**) Qualitative comparison of the trajectory envelopes with and without solute gradients. (**D**) The velocity pdf of the colloids shows that those in the attractive case experience a higher velocity in both the direction of flow and perpendicular to it. This effect is stronger in the disordered case due to the broader flow velocity distribution in the medium. All panels represent experimental data.

To understand how solute gradients affect particle dynamics, we track the mean velocity of the colloids within the field of view (white box in Fig. 1A) alongside their density. In the control case, the mean velocity decreases over time in proportion to the decreasing colloid density (black line and symbols in Fig. 3A). This trend does not reflect changes in background flow; rather, it arises because faster moving colloids exit the observation region first, leaving slower colloids behind and reducing the mean velocity at later times. In contrast, the presence of solute gradients disrupts this proportional relationship. For an attractive solute front, both the mean velocity and colloid density initially increase before eventually decreasing (blue line and symbols in Fig. 3A). For a repulsive solute front, the opposite trend occurs: The mean velocity and density initially decrease before rising at later times (cyan line and symbols in Fig. 3A).

To understand the mechanism behind these changes in the mean density and velocity of colloids, we probe the particle trajectories within the corresponding time window (Fig. 3A, double-headed arrow). We observe a decrease in the transverse dispersion of colloids in the repulsive case and an opposite effect in the attractive case (Fig. 3, B and C). The velocity probability density function (pdf) of the colloids shows those in the attractive case experience a higher velocity in both the direction of flow and perpendicular to it (Fig. 3D). Our observations clearly indicate the strong influence of solute gradients on the colloidal trajectories and velocities. However, it remains unclear whether these changes are due to the migration of colloids into/out of dead-end pores as expected by earlier works (*77*–*79*) or another mechanism modulates these changes. The impact of solute gradients on colloidal velocity statistics in ordered lattices without dead-end pores offers clues (Fig. 3D).

### Solute gradients drive the cross-streamline migration of colloids within the flow pathways

**Fig. 4.**
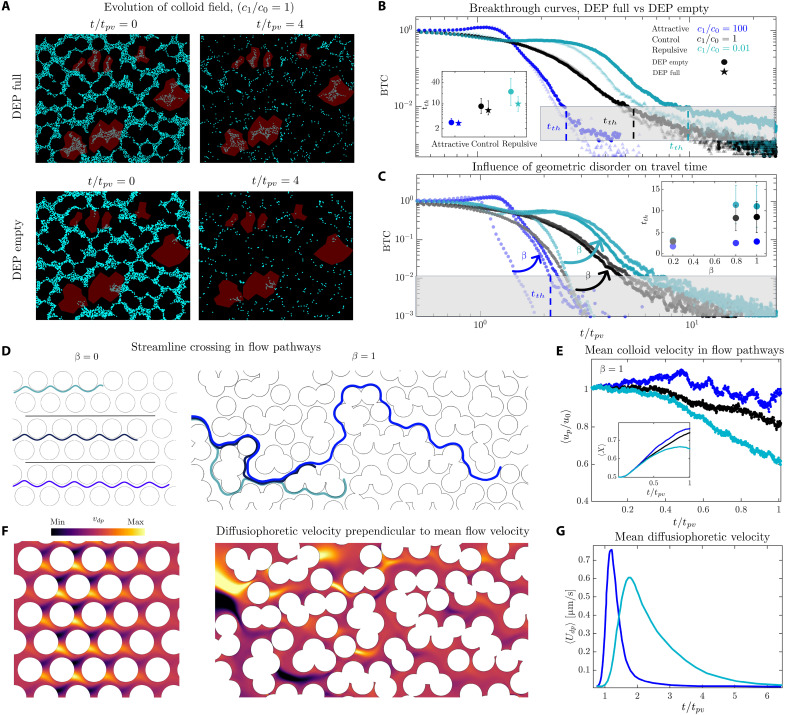
Solute gradients drive the cross-streamline migration of colloids within the flow pathways. (**A**) To disentangle the roles of dead-end pores and velocity heterogeneity, we compare experiments with dead-end pores filled (DEP full) or empty (DEP empty) of colloids. Breakthrough curves (BTCs) at the outlet (**B**) show that solute gradients strongly affect travel times ($t_{\mathrm{th}}$ defined when the BTC drops below a threshold value), but DEP full/empty cases evolve similarly (inset), indicating that macroscopic differences arise from phoretic cross-streamline migration within flow pathways rather than exchange with dead-end pores. (**C**) Attractive solute fronts weaken, whereas repulsive fronts strengthen, the effect of disorder on colloid transport (inset: $t_{\mathrm{th}}$ versus disorder strength β). (**D**) Simulations confirm drift toward slow/fast streamlines in repulsive/attractive cases. (**E**) This migration shifts mean velocities relative to control, accelerating or slowing travel (inset: mean position versus time). (**F**) Cross-streamline migration originates from solute gradients normal to flow, seen in $v_{\text{dp}}=\Gamma_{p}\text{∂}\text{ln}c/\text{∂}y$. (**G**) Although much weaker than pore flow velocities, phoretic drifts leave a substantial macroscopic footprint.

To disentangle the roles of dead-end pores from velocity heterogeneity in the medium, we performed experiments in which the dead-end pores were initially empty of colloids (Fig. 4A; Materials and Methods). We monitored the travel time of colloids through the medium using breakthrough curves, i.e., the particle flux measured directly downstream near the outlet. Although solute gradients strongly influence these breakthrough curves, we find that whether dead-end pores are initially filled or empty has little effect on their early-time evolution, as expected (Fig. 4B). This observation demonstrates that the pronounced macroscopic changes in colloid transport observed in the Fickian regime are not driven by phoretic migration into or out of dead-end pores but rather by the effect of solute gradients on colloid motion within the preferential flow pathways. This occurs despite flow velocities in these pathways being orders of magnitude larger than the phoretic velocities, i.e., O(100 μm/s) versus O(1 μm/s) (see Fig. 1B versus Fig. 4G). These experiments further reveal the interplay between diffusiophoresis and velocity heterogeneity. The impact of geometric disorder on flushing time, quantified by $t_{\text{th}}$ (Fig. 4B), is strongly suppressed under attractive solute gradients and slightly enhanced under repulsive gradients. Together, these results show that diffusiophoresis can modulate how velocity heterogeneity influences colloid transport.

To probe the impact of phoretic migration within the preferential flow pathways on colloid transport, we performed Lagrangian particle simulations with initially empty dead-end pores. These simulations reveal that particles originating from the same location can diverge due to phoretic effects (Fig. 4D). This cross-streamline migration, i.e., deviation from flow streamlines, produces a net increase in the mean colloid velocity under attractive solute gradients and the opposite effect under repulsive gradients (Fig. 4E), consistent with experimental trends (Fig. 3A). The parabolic flow profile in the preferential pathways, imposed by no-slip boundary conditions on the posts, generates local solute gradients that drive diffusiophoretic migration perpendicular to the flow (Fig. 4F). Although this cross-streamline migration is much slower than the mean flow velocity (Fig. 4G), it leaves a clear and measurable imprint on the macroscopic transport of colloids (Fig. 4, B and C). In section S7, we discuss how weak solute gradients perpendicular to the flow direction persist for times much larger than the solute diffusion timescale across a flow channel and how these weak gradients lead to cross-streamline migration.

### Flow disorder and solute gradients shape the macroscopic dispersion of colloids

**Fig. 5.**
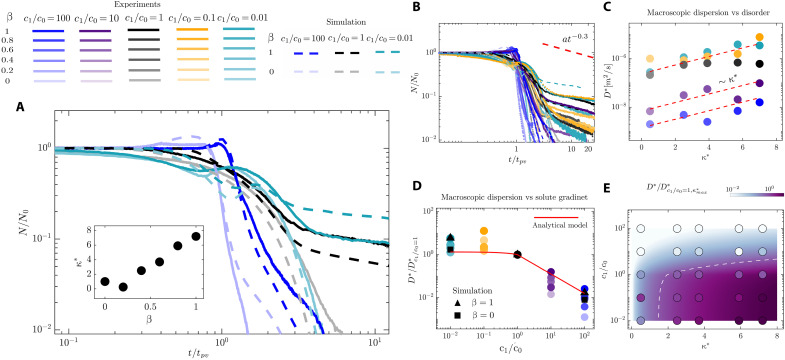
Flow disorder and solute gradients shape the macroscopic dispersion of colloids. (**A**) Time evolution of the normalized colloid density field in the experiments (solid lines) and simulations (dashed lines) corresponding to the ordered β = 0 and disordered β = 1 media and attractive $c_{1}/c_{0}=100$, control $c_{1}=c_{0}$, and repulsive $c_{1}/c_{0}=0.01$ cases. The inset shows the excess kurtosis in the transverse velocity distribution $\kappa^{*}$ as a function of the amplitude of geometric disorder β. (**B**) Data corresponding to 30 experiments and six simulations with different salt gradients, $0.01\leq c_{1}/c_{0}\leq 100$, and disorder strengths, 0≤β≤1. (**C**) The macroscopic dispersion coefficient obtained by fitting the ADE model to the Fickian regime shows an increasing trend with the velocity heterogeneity, characterized by excess kurtosis $\kappa^{*}$ (section S2). (**D**) The macroscopic dispersion coefficient in the attractive case decreases by two orders of magnitude compared to the control case, whereas it increases slightly in the repulsive case. We predict the influence of solute gradients on the cross-streamline migration within the flow pathways and its impact on the dispersion using a model of nondiffusive colloids in a channel flow (red line). (**E**) Regime diagram of macroscopic dispersion as a function of flow disorder and solute gradients, where circles represent the experimental data, the background color map represents a semiempirical model, and the dashed line is a representative contour line.

We conducted experiments across a broad range of solute gradients and geometric disorders, monitoring the evolution of the colloidal density field (Fig. 5, A and B). By fitting the early-time evolution of the density field to the solution of a one-dimensional (1D) advection-diffusion equation (section S4), we extracted the corresponding macroscopic dispersion coefficient for each experiment. We find that macroscopic dispersion increases with the degree of velocity heterogeneity in the medium (Fig. 5C). To quantify velocity heterogeneity, we use the excess kurtosis of the transverse velocity distribution, $\kappa^{*}$, which measures the “tailedness” of a distribution relative to a normal distribution (see section S2 for a discussion of other metrics that could be used for disorder characterization). We note that the slope of power law tail in the non-Fickian regime seems to be uninfluenced by the solute gradients, consistent with earlier predictions (*78*).

The influence of flow disorder, however, is modulated by the solute gradients. The dispersion decreases in the attractive case by almost two orders of magnitude compared to the control case and shows a modest increase in the repulsive case (Fig. 5D). These changes can be understood as direct consequences of cross-streamline migration effect within the flow pathways, leading to a net increase/decrease in colloid velocities in attractive/repulsive cases, respectively. A net increase in the colloidal velocities in the attractive case leads to the faster removal of colloids and, therefore, a smaller macroscopic dispersion, and the opposite effect is showed in the repulsive case.

To gain insight into the influence of solute gradients on the cross-streamline migration of colloids and their macroscopic dispersion, we develop a model for diffusiophoretic, nondiffusive colloids in a channel flow as an analog for a preferential flow pathway in a porous medium (section S7). In the absence of diffusion, colloids can only phoretically move across the streamlines due to solute gradients. The velocity gradient between these streamlines, in turn, leads to the dispersion of colloids in the flow direction. Our model predicts that the attractive solute front significantly decreases the colloidal dispersion $D^{*}/D_{\text{control}}^{*}\approx {\left(c_{1}/c_{0}\right)}^{-\Gamma_{p}/D_{s}}$ and the repulsive front slightly enhances it $D^{*}/D_{\text{control}}^{*}\approx \left[7\sqrt{3}-3{\left(c_{1}/c_{0}\right)}^{\Gamma_{p}/D_{s}}\right]/9$, qualitatively capturing the trend observed in our experiments (Fig. 5D). While capturing the impact of cross-streamline migration on dispersion in a channel, this simple model misses many important aspects of dispersion in a porous medium, including mechanical dispersion due to flow disorder (*102*–*106*).

The macroscopic dispersion of colloids through porous media is, therefore, modulated by both flow disorder and solute gradients as represented in the regime diagram of Fig. 5E. Here, the circles represent the experimental data, and the color map represents the semiempirical relationship $D^{*}\kappa^{*}$, where the empirical scaling with the flow disorder $\kappa^{*}$ is taken from Fig. 5C, and the dispersion $D^{*}$ is predicted using the model above.

## DISCUSSION

Our observations highlight the strong influence of phoretic migration on determining the dispersion and transport of colloids in porous and crowded environments. We demonstrated that solute gradients can strongly modulate the influence of geometric disorder on the transport and macroscopic dispersion, and suppress the non-Fickian regime. Unexpectedly, we observe that, even when the phoretic velocities are orders of magnitude weaker (≈1 μm/s) than the convective background flows (≈100 μm/s), they can change the macroscopic dispersion of colloids by orders of magnitude compared to the classical predictions. We explained this to be due to the substantial and long-lasting fingerprints of the phoretic streamline crossing in the flow pathways. The persistence of these trends in ordered and disordered flow fields aligns with reports on the signatures of diffusiophoretic migration on the macroscopic dispersion of colloids in cellular, chaotic, and even turbulent flows (*107*–*114*).

Our work suggests that diffusiophoresis may have implications in a variety of geophysical, environmental, and industrial scenarios where strong background flows are often present. In wastewater treatment facilities, chemical gradients can arise naturally or be engineered to modulate the transport and deposition of colloidal pollutants, affecting membrane fouling and overall filtration efficiency (*115*–*117*). In chromatography, controlled chemical gradients within porous media can be exploited to enhance the separation of complex colloidal mixtures, allowing for a more precise resolution of the target compounds (*118*–*120*). In agriculture, fertilizer and pesticide gradients can influence microplastic retention and migration, as well as the distribution of organic colloids rich in nutrients that are vital to soil health (*22*–*28*). Coastal and estuarine systems, characterized by strong salinity and nutrient gradients, present additional natural settings where diffusiophoresis could control the dispersion of both natural colloids and anthropogenic microplastics, influencing nutrient cycling and pollutant transport (*121*–*123*). Last, within biological tissues, diffusiophoretic forces could contribute to the targeted delivery of drug-carrying colloids by working in concert with interstitial fluid flows ensuring that therapeutics reach their intended sites with enhanced precision (*124*–*126*).

Classic descriptions of colloid transport in porous media consider geometric constraints, hydrodynamic interactions, and equilibrium forces that govern particle adhesion and detachment. In this framework, the influence of solutes is merely in determining the range of equilibrium and short-ranged, DLVO-type interactions between colloids and surfaces, and the macroscopic dispersion of colloids is described as a function of the mean flow velocity (*47*–*70*). Our observations point to the need for revisiting these classical descriptions to incorporate the nonequilibrium effects of solute gradients.

## MATERIALS AND METHODS

### Experimental methods

Microfluidic chips were fabricated using standard photolithography techniques, plasma bonding polydimethylsiloxane (PDMS) channels on glass substrates. We designed hexagonal lattices with obstacle/post diameter of 2*R* = 165 μm and set the spacing between the posts, i.e., pore size to *d*_*p*_ = 35 μm, and introduced disorder by randomly perturbing the location of the obstacles with an amplitude βL, where $L=2R+d_{p}$ and 0 ≤ β ≤ 1. The flow through the chip was fixed at 0.01 μl/s using a Harvard syringe pump. The images were recorded using a Nikon Ti-2 inverted microscope equipped with an ORCA-Fusion Digital CMOS camera with a spatial resolution of 2300 by 2300 pixels.

We used carboxylate-coated fluorescent polystyrene microspheres with a diameter of 1 μm with excitation and emission wavelengths of 540 and 560 nm, respectively. These particles are almost two orders of magnitude smaller than the typical pore size of 35 μm and channel height of 50 μm. The colloidal suspensions are very dilute with a volume fraction of 0.1%, leading to a packing fraction of ≈10^−3^. Note that the hydrodynamic disturbance velocity due to diffusiophoresis decays very fast as 1/*r*^3^ away from each particle in contrast to the 1/*r* decay due to the body force. We can therefore safely neglect the interactions between the particles and with the surfaces and consider them as point-like. We further conducted a few experiments using 10 times higher volume of fraction of colloids. The main trends observed in our experiments remained the same, suggesting that our experiments are in the dilute regime (section S8). A few theoretical works suggest that the diffusiophoretic mobility of colloids can change (increase/decrease) at high volume fractions, where polarization of the ions in the double layer around colloids, or the overlap between the diffuse layers become important (*127*). It would certainly be interesting to probe the role of colloidal volume fraction on the impact of diffusiophoresis in the future.

We added the colloids to an aqueous solution of lithium chloride with molarity in the range of 0.01 to 100 mM. Within this range of solute concentrations, Debye layer screening prevented any particle aggregation or exclusion in our experiments. The colloids we use are slightly denser than water (1050 kg/m^3^), leading to an estimated Stokes settling timescale of around 1 hour ($H/V_{s}$, where $V_{s}={\mathrm{gd}}^{2}\left(\rho_{p}-\rho_{l}\right)/\left(18\mu \right)\approx 20\;\text{nm}/s$). The typical timescale of our experiments is on the order of 10 to 20 min over which there is a constant flow in the main flow pathways. Although particles could slightly settle in the stagnant pockets, our observations indicate that the colloid density remains relatively uniform, likely due to size exclusion by the walls and Brownian motion as discussed in (*92*).

In the experiments reported in Fig. 4, the dead-end pores were not initially filled with colloids. To achieve this, we first injected the solute solution without colloids and subsequently introduced the colloids into the chip. The flushing experiments were started shortly after, well before colloids had time to diffuse into the dead-end pores, and therefore colloids remained confined to the convective pores only.

In a few experiments, we visualized the invading solute front by adding a dilute concentration (0.01 mM) of fluorescein sodium salt with excitation and emission wavelengths 460 and 515 nm, respectively. We note that the diffusivity of fluorescein is slightly different from the lithium chloride 4.2 × 10^−10^ m^2^/s versus 1.37 × 10^−9^ m^2^/s, and therefore its evolution can only be considered an approximate proxy for the evolution of the lithium chloride front. Furthermore, the presence of multiple ions could complicate the picture due to the coupled transport of ions (*96*, *128*, *129*). Therefore, we limited the use of fluorescein to a few representative experiments. We used dual-channel imaging with 10 frames/s to monitor the evolution of both colloids and the salt front in these experiments. All other experiments were done using a single-channel imaging.

To track the trajectories of the colloids, images were recorded at a rate of 64 frames/s, with a minimum particle displacement of 3 pixels per frame, covering a field of view measuring 2300 by 1800 pixels. We used an in-house code for the image processing in MATLAB together with the open-source software, TracTrac (*130*). The mean velocity at time $t_{k}$ is defined as $\bar{u}\left(t_{k}\right)=\frac{1}{N_{k}}\sum_{i=1}^{N_{k}}\left\|u_{i}\left(t_{k}\right)\right\|$, where $N_{k}$ is the number of tracked colloids at time step $t_{k}$, and $\left\|u_{i}\left(t_{k}\right)\right\|$ is the magnitude of the instantaneous velocity vector of the *i*th colloid at that time. To characterize the heterogeneity in the flow, we used the excess kurtosis of the transverse component of velocity field $\kappa^{*}=\kappa /\kappa_{\beta =0}$, which is a measure of the “tailedness” of a distribution relative to the normal distribution, $\kappa =\frac{\mu_{4}}{\sigma^{4}}-\kappa_{\text{normal}}$, where μ_4_ and σ are the fourth central moment and SD of the distribution and $\kappa_{\text{normal}}$ is the kurtosis of normal distribution (section S2).

### Diffusiophoretic mobility

The diffusiophoretic mobility of the colloids in a binary z-z electrolyte using the thin Debye layer approximation can be written as (*1*, *131*)

$$\Gamma_{p}=\frac{\epsilon }{\eta }{\left(\frac{k_{B}T}{\mathrm{ze}}\right)}^{2}\left(\underset{\text{electrophoresis}}{\underbrace{\beta_{s}\frac{\mathrm{ze}\zeta_{p}}{k_{B}T}}}+\underset{\text{chemiphoresis}}{\underbrace{4\text{lncosh}\left(\frac{\mathrm{ze}\zeta_{p}}{4k_{B}T}\right)}}\right)$$ (1)

where η is the viscosity of the fluid, *T* is the temperature, ϵ is the permittivity of the medium, $k_{B}$ is the Boltzmann constant, *z* is the electrolyte valence, *e* is the elementary charge, $\zeta_{p}$ is the zeta potential of the colloid, and $\beta_{s}=\left(D_{+}-D_{-}\right)/\left(D_{+}+D_{-}\right)$ is the mobility difference of cation and anion, characterizing the electrophoretic strength. For LiCl, $D_{+}=1.03\times 10^{-9}$ m^2^/s, and $D_{-}=2.03\times 10^{-9}$ m^2^/s. The colloids used in our experiments have a zeta potential $\zeta_{p}\approx -70\;\text{mV}$ (*92*, *99*, *132*), leading to the diffusiophoretic mobility of $\approx \Gamma_{p}=800$ μm^2^/s. Although the zeta potential depends on the solute concentration, pH value, and counter-ion type (*133*, *134*), we assume the zeta potential to be a constant in all the experimental scenarios for simplicity. From the linearized Poisson-Boltzmann equation, we can then obtain the electrostatic potential at a distance *r* from charged spherical particle of radius *a* as $\phi \left(r\right)=\zeta_{p}\left(a/r\right)e^{-\left(r-a\right)/\lambda_{D}}$, where the Debye layer thickness, $\lambda_{D}=\sqrt{\epsilon k_{B}T/\left(2n^{0}e^{2}\right)}$, sets the range of interactions with $n^{0}$ as the bulk concentration of the ions. In our experiments, this length is O(10 nm), which is much smaller than the particle diameter of 1 μm.

### Numerical simulations

We numerically solve the following equations using the open-source software OpenFOAM, which uses finite volume (FV) schemes for the discretization of partial differential equations

$$\begin{array}{c} 0=-\tilde{\nabla }\tilde{p}+{\tilde{\nabla }}^{2}\tilde{u}\\ 0=\tilde{\nabla }\cdot \tilde{u} \end{array}$$ (2)

$$\frac{\text{∂}c}{\text{∂}\tilde{t}}=\frac{1}{{\text{Pe}}_{s}}{\tilde{\nabla }}^{2}c-\tilde{u}\cdot \tilde{\nabla }c$$ (3)

$$\frac{\text{∂}n}{\text{∂}\tilde{t}}=\frac{1}{{\text{Pe}}_{p}}{\tilde{\nabla }}^{2}n-\tilde{\nabla }\cdot \left[\left(\tilde{u}+{\tilde{u}}_{\mathrm{dp}}\right)n\right]$$ (4)

where $\tilde{p}=p/\left(\mathrm{\mu U}/\lambda \right)$ is the nondimensional fluid pressure with μ as the fluid viscosity, *U* as the characteristic flow velocity (mean velocity), and λ as the characteristic lengthscale (pore size), $\tilde{u}=u/U$ is the nondimensional flow velocity, $\tilde{t}=t/\left(\lambda /U\right)$, and *c* and *n* are the solute and colloid density, respectively. The Péclet numbers for the solute and particles are defined as ${\text{Pe}}_{s}=\mathrm{U\lambda}/D_{s}$ and ${\text{Pe}}_{p}=\mathrm{U\lambda}/D_{p}$, where $D_{s}$ and $D_{p}$ are the corresponding diffusion coefficients. The diffusiophoretic velocity is defined as ${\tilde{u}}_{\mathrm{dp}}=u_{\mathrm{dp}}/U=\left(\Gamma_{p}/D_{s}\right)\left(1/{\text{Pe}}_{s}\right)\tilde{\nabla }\text{ln}c$, where $\Gamma_{p}$ is the phoretic mobility of the colloids.

The quasi-2D meshes were created with the mesh generators snappyHexMesh and extrudeMesh, generating 13.5 million cells in our simulation domain. The steady flow field was first solved using the simpleFoam solver. Then, the transient transport processes of the solute and colloidal particles were computed with our customized solver, which considered the diffusiophoresis effect on particles. The initial solute concentration and particle density were set as 1 in the whole domain. At the inlet, the solute concentration was set to 0.01, 1, and 100, respectively for the repulsive, control, and attractive cases, whereas the particle density was set to 0. Zero-gradient boundary conditions were used at the outlet for both the scalars. In the gap direction, the symmetry boundary condition was used at the upper boundary. The other boundaries were treated as no-slip and no-flux walls. A typical simulation corresponding to ~2000 s in the simulation time is performed on the Yale High Performance Computing Cluster on 450 CPUs for ~5 days. We neglect the role of diffusioosmosis in our numerical simulations as accounting for them leads to the coupling of solute and velocity fields, resulting in prohibitively high computational costs. Closer inspection of our experimental observations further shows no clear sign of diffusioosmotic flows. The qualitative agreement between our numerical simulations and experimental observations suggests that diffusioosmosis might only have a weak effect in our system. Recent works have demonstrated that diffusioosmotic flows could affect the transport of colloids both quantitatively and qualitatively (*5*, *98*–*100*, *135*–*143*). Therefore, it would be interesting to probe the impact of diffusioosmosis on the transport of colloids in porous media flows in the future.

## Supplementary Materials

### This PDF file includes:

Section S1 to S8 Figs. S1 to S12 References
