# Supplementary material for: Diffusiophoretic transport of colloids in porous media
Source: Sci Adv. 2026 Feb 11;12(7):eady9874. doi: 10.1126/sciadv.ady9874 (PMC12893296; doi:10.1126/sciadv.ady9874)
Supplement: Supplementary file 1 — Section S1 to S8 Figs. S1 to S12 References [file sciadv.ady9874_sm.pdf]

Science Advances
AAAS

Supplementary Materials for

Diffusiophoretic transport of colloids in porous media

Mobin Alipour *et al.*

Corresponding author: Amir A. Pahlavan, amir.pahlavan@yale.edu

Sci. Adv. **12**, eady9874 (2026)
DOI: 10.1126/sciadv.ady9874

This PDF file includes:

Section S1 to S8
Figs. S1 to S12
References

1. Geometric characteristics of the medium

To characterize the pore size distribution of the medium, we first detect equidistant lines passing through the channels, and define the local pore size at each point based on the diameter of the fitted circles along these lines (Fig. S1 (a)). The pore size distribution broadens as the strength of the disorder parameter β increases (Fig. S1 (b)). Introducing the geometric disorder leads to small changes in the medium porosity, increasing it from 0.36 to 0.38 due to the overlapping of the obstacles (Fig. S1 (c)). The mean pore size, however, shows a much stronger dependence on β, increasing from around 40 microns to 60 microns, i.e., a 50 percent increase (Fig. S1 (d)). The increase in the mean pore size in turn leads to the emergence of preferential flow pathways shown in Fig. 1 of the main text.

An alternative way of characterizing the impact of disorder on the geometric features of the medium is to probe the local porosity. We define the local porosity over a window size of the same area as one lattice unit; we then move this window in the horizontal and vertical directions by half the size of the lattice, scanning the entire medium (Fig. S1 (e)). The distribution of the local porosities obtained then provide a measure of the disorder in the system (Fig. S1 (f)), showing an increasing variance as a function of the disorder amplitude β (Fig. S1 (g)).

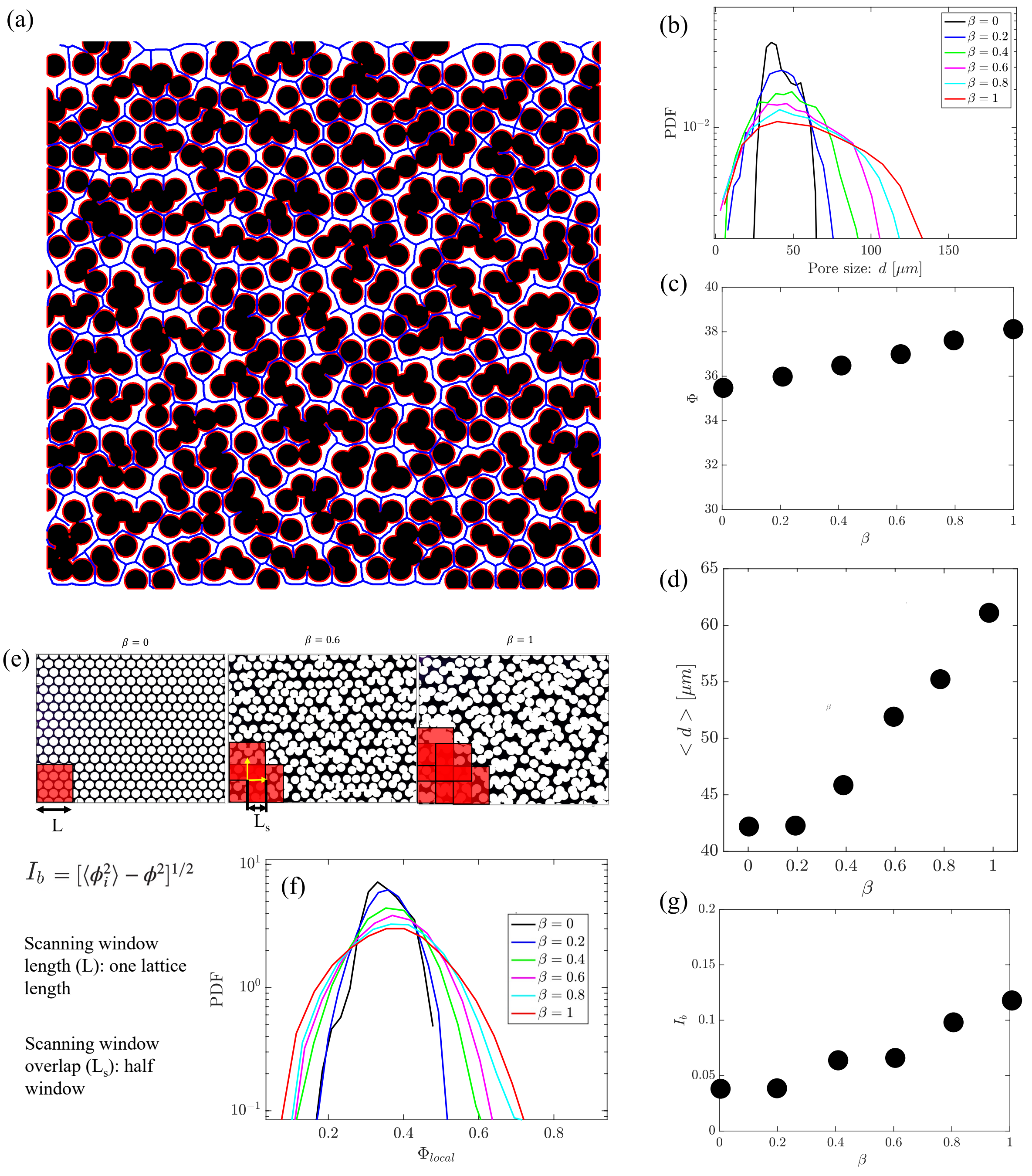

FIG. S1. (a) The equidistant lines between posts are detected and then pore size is calculated based on the diameter of a best fitted circle. Panel (b) shows the pore size distribution as a function of disorder. (c) While porosity is weakly affected by the disorder due to the overlapping posts, the mean pore size shows a strong dependence on the disorder strength β (d). (e) Local porosity Φ_{local} is calculated by moving a window of the size of one lattice unit. (f) The PDF of the local porosities broadens as the strength of the disorder increases as also evident from the variance of the local porosity field (g).

2. Flow velocity statistics

To ensure the homogeneity of our disordered media over the length of the chip, we probed the velocity statistics over different windows along the medium. The distribution of velocity magnitude U, the horizontal velocity component u, and local orientation of particle trajectories defined as $\theta = atan(v/u)$ all show similar trends, overlaying on each other (Fig. S2).

We further characterize the excess kurtosis of the vertical v component of the velocity and the fraction of the stagnant pockets over the different windows along the medium (Fig. S3). The excess kurtosis of the transverse component of velocity field $\kappa^* = \kappa/\kappa_{\beta=0}$ is a measure of the "tailedness"

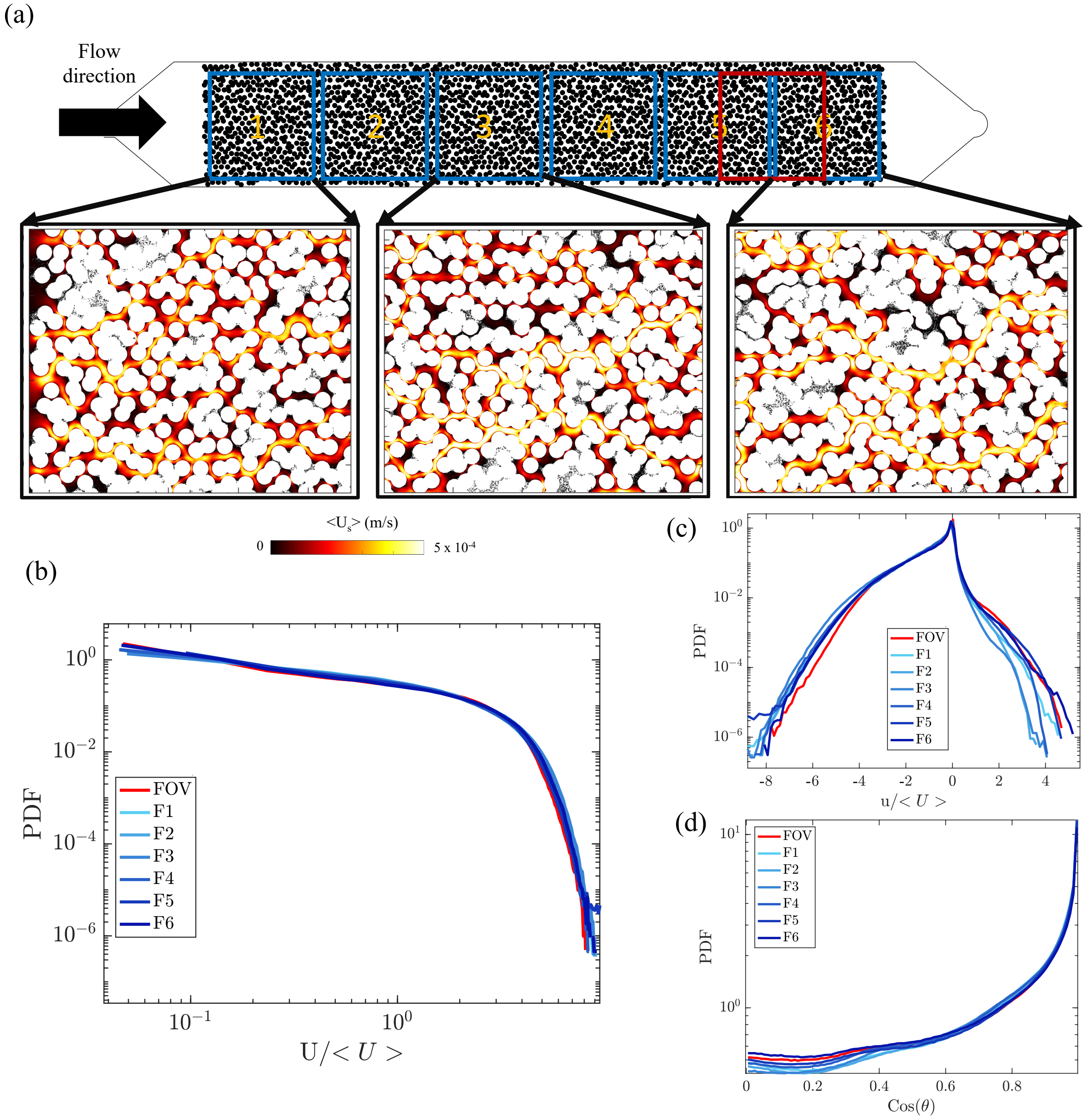

FIG. S2. The velocity statistics over different windows along the medium (a) show similar distribution, ensuring the homogeneity of the medium and absence of long-range correlations (b-d).

of a distribution relative to the normal distribution, $\kappa = \frac{\mu_4}{\sigma^4} - \kappa_{\text{normal}}$, where μ_4 and σ are the fourth central moment and standard deviation of the distribution and κ_{normal} is the kurtosis of normal distribution. We note that the excess kurtosis shows a non-monotonic trend at small disorder strength β (Fig. S3 (c)). This is consistent with the recent observations of [147] on the non-monotonic increase of longitudinal dispersion with the degree of disorder in the medium. Here, this non-monotonic behavior is due to the fact that our ordered medium is an aligned hexagonal array of obstacles with a higher probability of low velocity zones between these obstacles (Fig. S3 (b)). Introducing weak disorder removes these stagnant pockets, and brings the velocity distribution closer to that of the Gaussian distribution. In a staggered array, however, this non-monotonic trend will not be present [81].

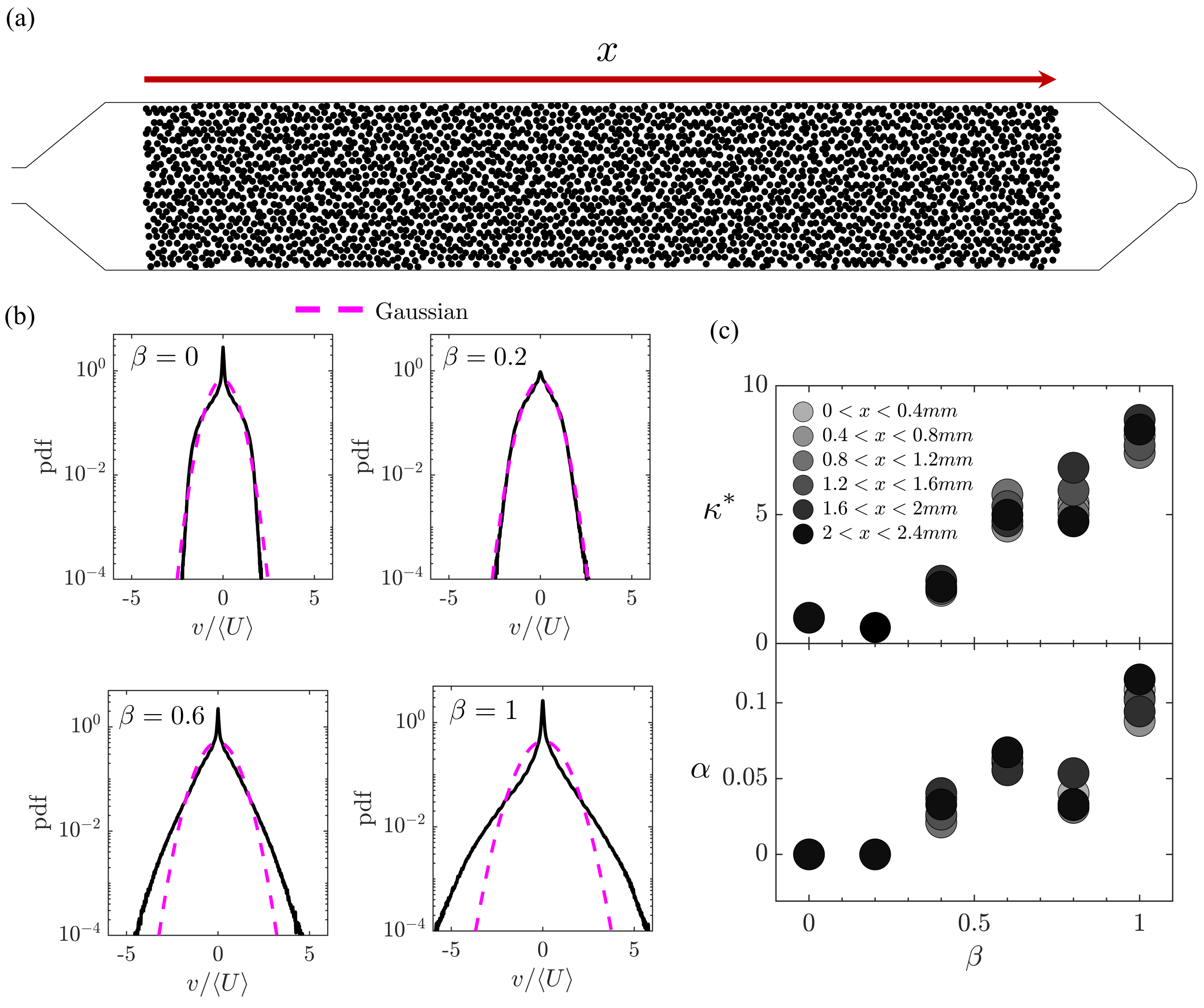

FIG. S3. (a) Microfluidic chip. (b) pdf of the transverse velocity distribution and the corresponding Gaussian fits for four different disorder strengths. (c) The normalized excess kurtosis of the transverse velocity component $\kappa^* = \kappa/\kappa_{\beta=0}$ and the area fraction of stagnant pockets α as a function of the disorder strength β probed over different windows along the medium.

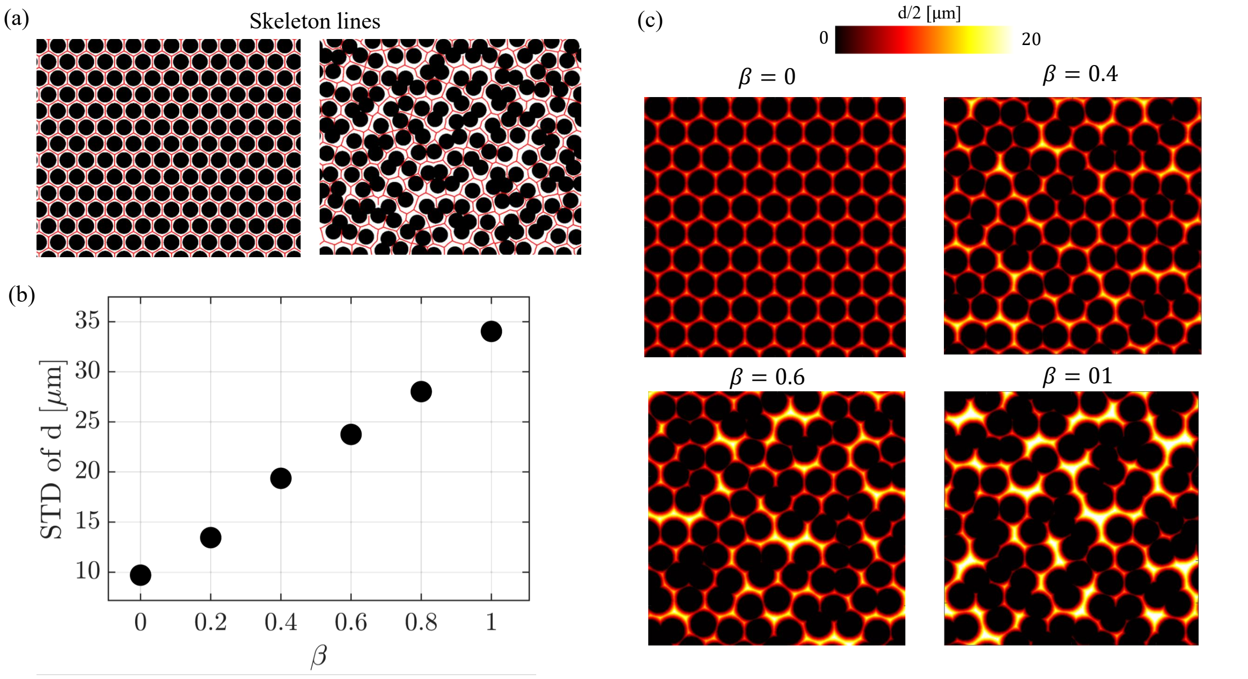

FIG. S4. The standard deviation of the pore size distribution increases linearly with the amplitude of geometric disorder β (a,b). (a) The skeleton lines used to define the minimum distances of the posts to the line, $d/2$. (b) Standard deviation of the pore diameter d. (c) Visual representation of the pore size $d/2$ field for 4 different disorder strength.

Another metric to quantify the influence of geometric disorder is the standard deviation of the pore size distribution (Fig. S4). This metric shows a linear trend with the amplitude of geometric disorder β. We define the normalized standard deviation $\tilde{\sigma}_d = \sigma_d/\mathrm{d}_p$, where d_p=35 μm is the minimum pore size in the ordered case. This normalized standard deviation spans $0.3 < \tilde{\sigma}_d < 1$.

3. Influence of salt type

All experiments reported in the main text were performed using LiCl, which raises the question of whether our observations generalize to other salts. The effect of salt type on the diffusiophoretic transport of colloids has been investigated in numerous studies, e.g., [92, 96, 144, 146]. Differences in cation and anion diffusivities influence the diffusiophoretic mobility of colloids. For a binary z−z electrolyte, under the thin Debye layer approximation, the mobility can be expressed as [1,131]:

$$\Gamma_p = \frac{\varepsilon}{\eta}\left(\frac{k_B T}{ze}\right)^2 \left(\underbrace{\beta_s \frac{ze\zeta_p}{k_B T}}_{\text{electrophoresis}} + \underbrace{4\ln\cosh\left(\frac{ze\zeta_p}{4k_B T}\right)}_{\text{chemiphoresis}} \right), \quad (1)$$

where η is the viscosity of the fluid, T is the temperature, ε is the permittivity of the medium, k_B is the Boltzmann constant, z is the electrolyte valence, e is the elementary charge, ζ_p is the colloid zeta potential and $\beta_s = (D_+ - D_-)/(D_+ + D_-)$ is the mobility difference of cation and anion, characterizing the electrophoretic strength. For LiCl, $D_+ = 1.03 \times 10^{-9} \mathrm{m}^2/\mathrm{s}$, $D_- = 2.03 \times 10^{-9} \mathrm{m}^2/\mathrm{s}$, leading to $\beta_s = -0.33$. For other monovalent salts such as NaCl and KCl, we have $\beta_s = -0.20$ and $\beta_s = -0.02$, respectively [132]. Therefore, the diffusiophoretic mobility corresponding to LiCl, NaCl and KCl, can be estimated to be $\approx 800, 650, 420 \times 10^{-12} \mathrm{m}^2/\mathrm{s}$, respectively.

To assess the influence of salt type on our observations, we repeated attractive experiments on $\beta = 1$ chips using NaCl and KCl (Fig. S5). We expect that a higher diffusiophoretic mobility will correspond to a stronger effect of the solute gradients on the evolution of the colloid field. Indeed, this is what we observe: LiCl produces the strongest impact on colloid removal, whereas KCl has the weakest. Thus, although the qualitative trends remain similar across salts, the salt type clearly affects the magnitude of diffusiophoretic transport. Exploring this influence systematically would be an interesting direction for future work.

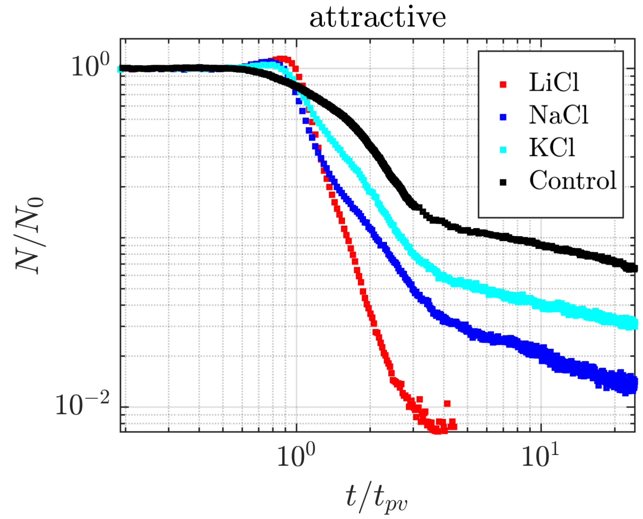

FIG. S5. Influence of salt type (LiCl, NaCl, KCl) on the evolution of colloid density field in the attractive case ($c_1/c_0 = 100$) in disorderd chips ($\beta = 1$).

4. 1D Advection-diffusion equation

To extract the macroscopic dispersion of colloids, D*, we used the 1-D advection diffusion equation:

$$\partial_{\bar{t}} N + \partial_{\bar{x}} N - \bar{D}^* \partial_{\bar{x}}^2 N = 0, \tag{2}$$

where $\bar{x} = x/L$, $\bar{t} = t/\tau_{\mathrm{PV}}$, $\bar{D}^* = D^*/(U_{\mathrm{x}} L)$. The solution of this equation is:

$$N(\bar{x}, \bar{t}) = \frac{1}{2}\left(1 + \mathrm{erf}\left(\frac{\bar{x} - \bar{t}}{\sqrt{4\bar{D}^* \bar{t}}}\right)\right). \tag{3}$$

Therefore, the colloid flux can be defined as:

$$\bar{F} = N(\bar{x}, \bar{t}) - \bar{D}^* \partial_{\bar{x}} N = 1 - \frac{1}{2}\mathrm{erfc}\left(\frac{\bar{x} - \bar{t}}{\sqrt{4\bar{D}^* \bar{t}}}\right) - \frac{1}{2}\sqrt{\frac{\bar{D}^*}{\pi \bar{t}}}\exp\left(-\left(\frac{\bar{x} - \bar{t}}{\sqrt{4\bar{D}^* \bar{t}}}\right)^2\right). \tag{4}$$

In breakthrough experiments, we record the particle density at the outlet and use it as a proxy for the flux, thereby neglecting the diffusive contribution. Fixing $\bar{x} = 1$ (the outlet position) and introducing a temporal offset $\bar{t}_0$ to align the breakthrough curves (BTCs), we fit the data with

$$\bar{F}(\bar{t}) = 1 - \frac{1}{2}\mathrm{erfc}\left[\frac{1 - (\bar{t} - \bar{t}_0)}{\sqrt{4\bar{D}^*(\bar{t} - \bar{t}_0)}}\right],$$

where $\bar{t}_0$ and $\bar{D}^*$ are treated as free parameters. The best fits, shown in Fig. S6 (a), give $\bar{D}^*_{\mathrm{C}} \approx 0.07$, $\bar{D}^*_{\mathrm{R}} \approx 0.05$, and $\bar{D}^*_{\mathrm{A}} \approx 0.002$ for the control, repulsive, and attractive cases, respectively. Here, $U_{\mathrm{x}} = 105\,\mu\mathrm{m/s}$, $L = 2.27\,\mathrm{cm}$.

We also monitored the mean particle density inside the field of view (FOV), which spans $\bar{x} \in [0.7, 0.88]$. Integrating equation (3) over this interval yields

$$N_{\mathrm{FOV}}(\bar{t}) = \frac{1}{2} + \frac{1}{2(\bar{x}_2 - \bar{x}_1)}\Bigg[(\bar{x}_2 - \bar{t} + \bar{t}_0)\,\mathrm{erf}\Bigg(\frac{\bar{x}_2 - \bar{t} + \bar{t}_0}{\sqrt{4\bar{D}^*(\bar{t} - \bar{t}_0)}}\Bigg) - (\bar{x}_1 - \bar{t} + \bar{t}_0)\,\mathrm{erf}\Bigg(\frac{\bar{x}_1 - \bar{t} + \bar{t}_0}{\sqrt{4\bar{D}^*(\bar{t} - \bar{t}_0)}}\Bigg)$$
$$+ \sqrt{\frac{4\bar{D}^*(\bar{t} - \bar{t}_0)}{\pi}}\Bigg(e^{-\frac{(\bar{x}_2 - \bar{t} + \bar{t}_0)^2}{4\bar{D}^*(\bar{t} - \bar{t}_0)}} - e^{-\frac{(\bar{x}_1 - \bar{t} + \bar{t}_0)^2}{4\bar{D}^*(\bar{t} - \bar{t}_0)}}\Bigg)\Bigg]. \tag{5}$$

Figure S6 shows the fits to the experimental data for $\beta = 1$ in both FOV and BTC, from which the macroscopic dispersion values reported in the main text are inferred. Fitting the experimental $N_{\mathrm{FOV}}(\bar{t})$ data with this expression leads to $\bar{D}^*_{\mathrm{C}} \approx 0.07$, $\bar{D}^*_{\mathrm{R}} \approx 0.03$, and $\bar{D}^*_{\mathrm{A}} \approx 0.004$ for the control, repulsive, and attractive cases, respectively (Fig. S6 (b)). These values are close to the values obtained from the breakthrough curves, and show the same qualitative trends, indicating that we can rely on the dispersion coefficients extracted from the evolution of colloid density within the field of view to monitor the influence of solute gradients.

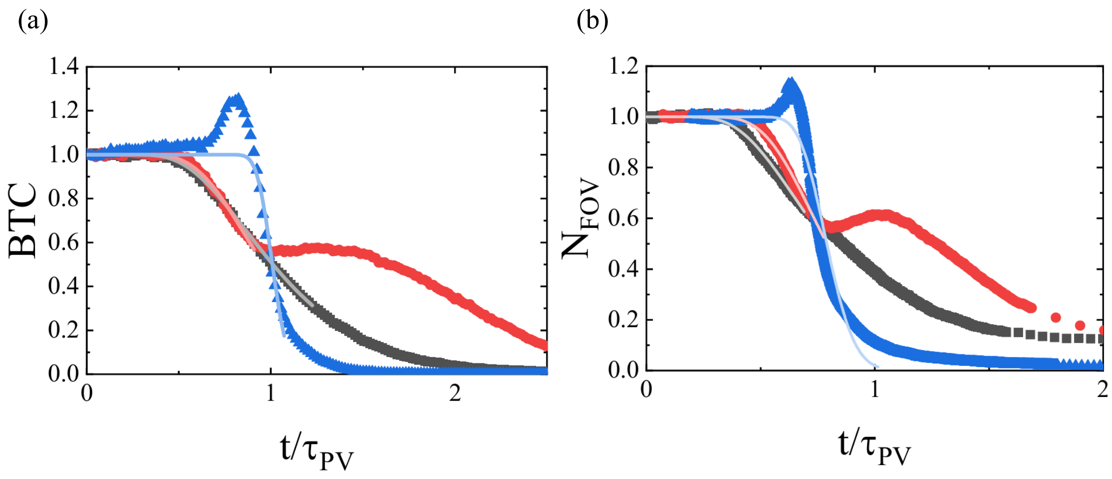

FIG. S6. The fitting of 1D ADE to the breakthrough curves (a), and field of view data (b) for $\beta = 1$ experiments. The black, blue, and red represent control, attractive, and repulsive cases, respectively.

5. Solute mixing and dispersion

The geometric disorder leads to the flow disorder, broadening the velocity field, creating high-velocity preferential flow pathways surrounding the low-velocity stagnant pockets. This velocity heterogeneity in turn leads to the non-uniform mixing of the solute front as evident from the evolution of the fluorescent dye in our experiments (Fig. S7 (a,c)). Disorder therefore leads to the persistence of solute gradients in the medium, driving the phoretic migration of colloids.

Experiments: We characterize the evolution of the solute mixing using the normalized light intensity $I/I_{\max}$, which evolves from zero before the fluorescent dye arrives in the field of view to one when the field of view is saturated with the dye (Fig. S7 (d)). We further define the variance of the light intensity $\sigma = \langle I^2 \rangle - \langle I \rangle^2$ as a measure of mixing. In the ordered geometry, this quantity reaches its peak and then quickly decays to zero as the solute front remains uniform. In the disordered geometry, however, both the increase and decay slow down. The increase to the peak slows down due to the non-uniform arrival of the solute front, which first enters the domain via the high-velocity pathways. The slow decay is due to the persistence of gradients around the stagnant fluid pockets, where diffusion is the only mechanism for mixing. Disorder prolongs this transition.

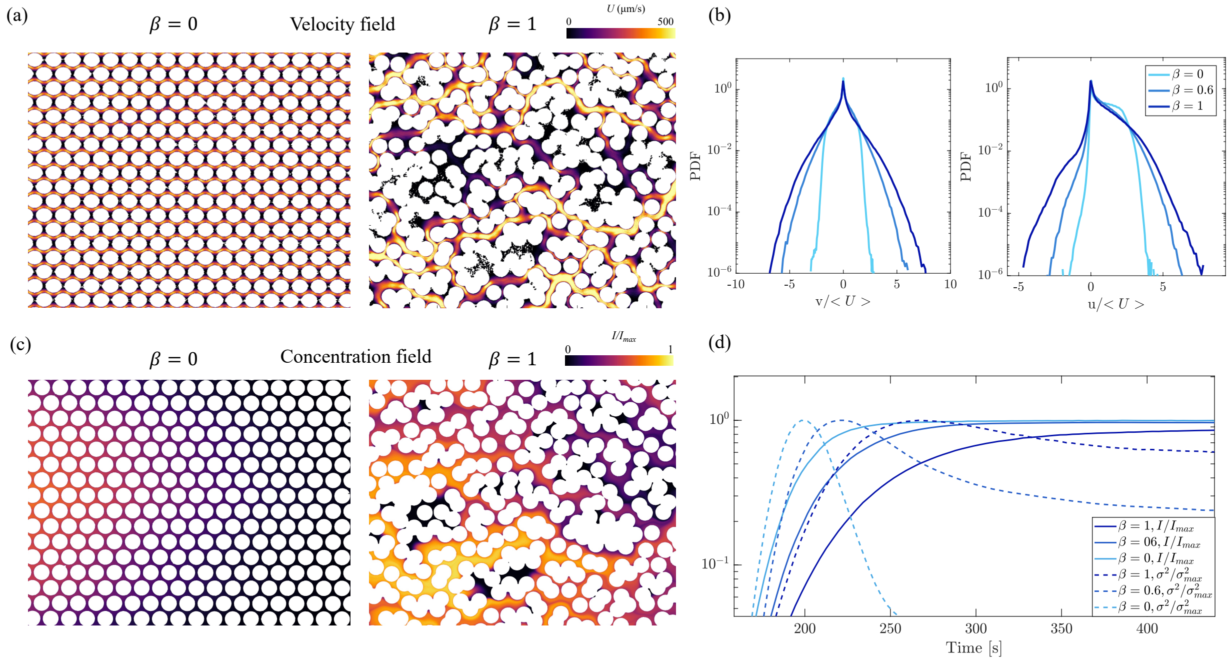

FIG. S7. (a, c) The velocity field and instantaneous salt concentration fields for ordered and disordered media. Disorder broadens the velocity field (b), leading to (d) the non-uniform evolution of the solute front, increasing its dispersion, and persistent gradients as characterized by the variance of the light intensity $\sigma^2 = \langle I^2 \rangle - \langle I \rangle^2$.

(a)

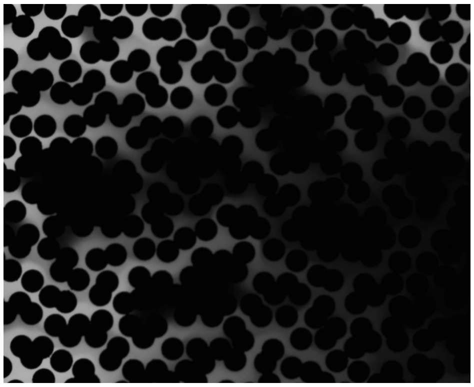

(b)

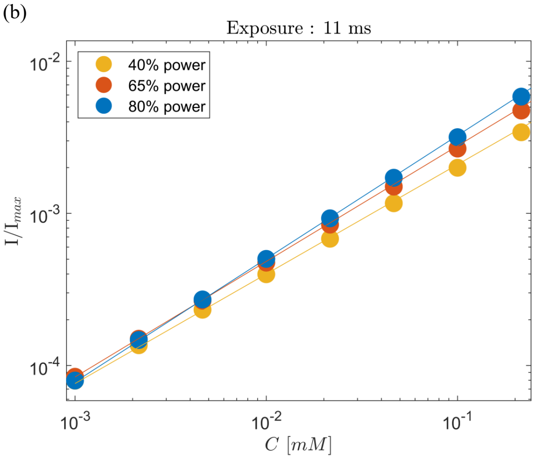

FIG. S8. (a) Snapshot of the fluorescein dye front. (b) Calibration of fluorescein dye against concentration shows a linear trend with no self-quench or saturation within our explored range. The trend is further not sensitive to the laser power within this range.

We have calibrated the light intensity against the concentration of fluorescein, demonstrating that within the range uses in our experiments, a linear trend exists and no saturation or self-quenching is observed (Fig. S8).

Numerical Simulations: We can gain further insight into the evolution of the solute field through the medium using numerical simulations (Fig. S9). The solute front advected through the porous medium also gets dispersed due to shear and flow heterogeneities; this diffuse solute front introduces another timescale T, which can be much larger than the solute diffusion timescale along a small dead-end pore. To define the timescale associated with the dispersion of the diffuse solute front, we can fit the solute front using the solution of a 1D advection-diffusion equation:

$$c(t) = c_0 + \frac{c_1 - c_0}{2}\left(1 + \mathrm{Erf}\left(\frac{U_\mathrm{x} t - x_\mathrm{FOV}}{\sqrt{4 D_\mathrm{eff} t}}\right)\right), \tag{6}$$

from which we can define the characteristic solute transition time as:

$$\tau_\mathrm{erf} = \frac{2\sqrt{4 D_\mathrm{eff}(x_\mathrm{FOV}/U_\mathrm{x})}}{U_\mathrm{x}}. \tag{7}$$

Our simulations (Fig. S9 (b)) show that this timescale can be $5-10$ times that of solute diffusion along a typical dead-end pore, leading to the solute gradients to persist in dead-end pores for much longer times than one otherwise expect from a sharp solute front. This dispersion of the solute front is associated with a decrease in the magnitude of diffusiophoretic velocity, but prolonging its effect (dashed lines in Fig. S9 (b)).

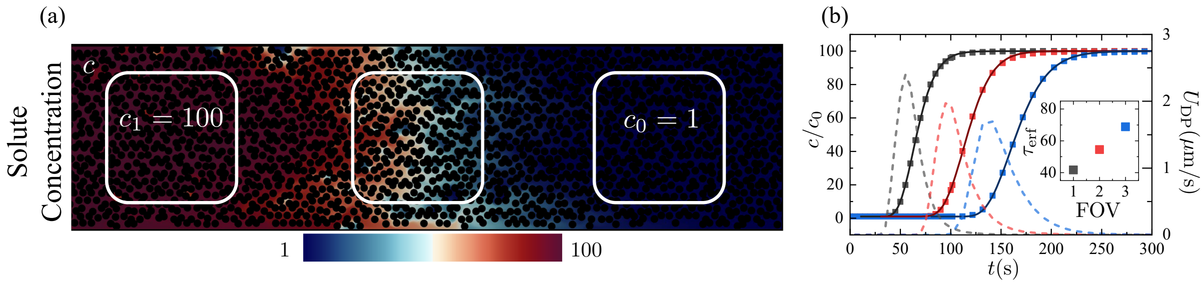

FIG. S9. (a) Dispersion fo solute front in heterogeneous medium. (b) Averaged solute concentration is shown in 3 different FOV as function of time. The diffusiophoretic velocity is plotted in dashed lines and the timescale obtained via fitting the error function to c/c_o is plotted in the inset.

6. Diffusiophoretic transport in a 1D dead-end pore

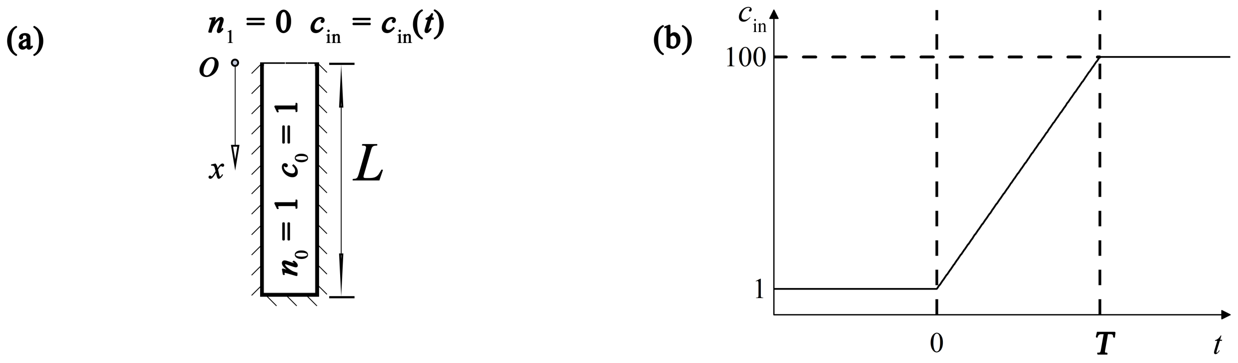

FIG. S10. (a) Illustration of the dead-end pore setup. (b) Linearly changing inlet solute concentration.

We consider a 1D dead-end pore with length L and inlet at $x = 0$, as shown in Fig. S10 (a). The influence of the flow is assumed negligible in the dead-end pore, which is true for dead-end pores with large aspect ratios, so that we can focus on the diffusiophoretic motion of particles induced by the solute diffusion process. The initial solute concentration and particle density are $c_0 = 1$ and $n_0 = 1$, respectively. And at the inlet, the boundary conditions are $c_1 = c_1(t)$ and $n_1 = 0$. Since solute front is dispersed in porous media flow, the dead-end pores in the media experience a smooth inlet solute concentration transition instead of a sharp step-function change. Therefore, to mimic a smooth inlet solute concentration profile, we follow the work of Migacz et al. [101] and impose a linearly varying inlet solute concentration profile $c_1(t)$ which increases from c_0 to c_1 over the duration T, as shown in Fig. S10 (b). The evolution of the solute field is governed by the equation:

$$\frac{\partial c(x,t)}{\partial t} = D_{\text{s}} \frac{\partial^2 c(x,t)}{\partial x^2}, \tag{8}$$

with the initial condition $c(x,0) = c_0$ and boundary conditions:

$$c(0,t) = \left\{ \begin{array}{c} c_0 + \frac{c_1 - c_0}{T} \cdot t,\ t \in [0,T) \\ c_1,\ t \in [T,\infty] \end{array} \right\}, \qquad \frac{\partial c}{\partial x}(L,t) = 0, \tag{9}$$

where D_{s} is the solute diffusivity. Upon non-dimensionalizing the above equations by introducing $\tau_{\text{s}} = \frac{L^2}{D_{\text{s}}}$, $\bar{x} = x/L$, $\bar{t} = t/\tau_{\text{s}}$, $\bar{T} = T/\tau_{\text{s}}$, $\bar{c} = \frac{c - c_0}{c_1 - c_0}$, we can obtain the analytical expression for the evolution of the solute field:

$$\bar{c}(\bar{x},\bar{t}) = \left\{ \begin{array}{l} \sum\limits_{n=0}^{\infty} \frac{2}{\lambda_n^3 \bar{T}} e^{-\lambda_n^2 \bar{t}} \sin(\lambda_n \bar{x}) + \frac{1}{2\bar{T}} \left(\bar{x}^2 - 2\bar{x}\right) + \frac{\bar{t}}{\bar{T}},\ \bar{t} \in [0,\bar{T}] \\ -\sum\limits_{n=0}^{\infty} \frac{2}{\lambda_n^3 \bar{T}} \left(1 - e^{-\lambda_n^2 \bar{T}}\right) e^{-\lambda_n^2 (\bar{t} - \bar{T})} \sin(\lambda_n \bar{x}) + 1,\ \bar{t} \in (\bar{T},\infty) \end{array} \right. \tag{10}$$

where $\lambda_n = \frac{(2n+1)\pi}{2}$. Since particle diffusion time across a typical dead-end pore is much larger than our experimental time, we further neglect particle diffusion. Therefore, the transport of each

non-diffusive particle is simply driven by the diffusiophoretic velocity, and its trajectory $\bar{x}(\bar{\xi}_0,\bar{t})$ governed by:

$$\frac{\partial \bar{x}\left(\bar{\xi}_0,\bar{t}\right)}{\partial \bar{t}} = \bar{U}_{\mathrm{DP}}\left(\bar{x},\bar{t}\right) = \bar{\Gamma}_{\mathrm{p}} \frac{\partial \ln\left(\bar{c}\left(\bar{x},\bar{t}\right) + \frac{1}{\beta - 1}\right)}{\partial \bar{x}}, \tag{11}$$

where $\bar{\xi}_0$ is the particle initial position, $\bar{\Gamma}_{\mathrm{p}} = \Gamma_{\mathrm{p}}/D_{\mathrm{s}}$ is the normalized diffusiophoretic mobility of the particle and $\beta = c_1/c_0$ is the ratio between the final and initial solute concentration. Consistent with the work of Migacz et al. [101], when $\bar{T} \to \infty$, the final particle position after the solute front passes can be derived:

$$\bar{x}_{\mathrm{F}}(\bar{\xi}_0) = \max(0, 1 - \left(1 - \bar{\xi}_0\right)\beta^{\bar{\Gamma}_{\mathrm{p}}}). \tag{12}$$

Therefore, only particles with initial position $\bar{\xi}_0 > 1 - \beta^{-\bar{\Gamma}_{\mathrm{p}}}$ will stay in the dead-end pore. Given that initially particles are uniformly distributed in the dead-end pore, we conclude that the proportion of the remaining particle is $\left(\frac{c_1}{c_0}\right)^{-\Gamma_{\mathrm{p}}/D_{\mathrm{s}}}$. Hence, for a porous medium with a stagnant area fraction α, we predict that after the solute front passes, the proportion of the particles remaining in these stagnant pockets is

$$N_1/N_0 \approx \alpha \left(c_1/c_0\right)^{-\Gamma_{\mathrm{p}}/D_{\mathrm{s}}}. \tag{13}$$

7. Diffusiophoretic transport in a 2D channel flow

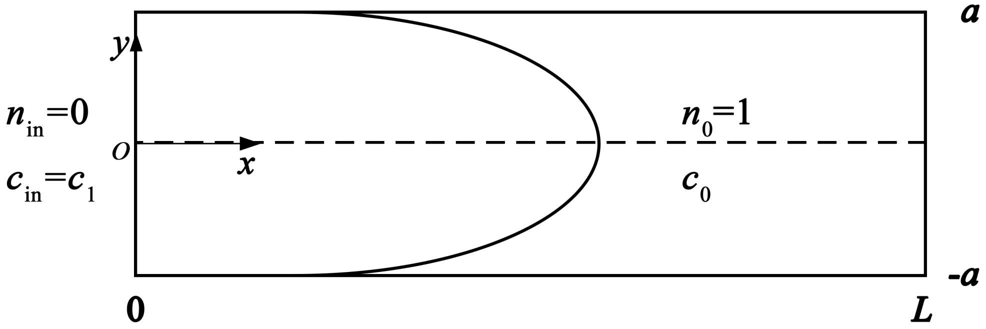

FIG. S11. Illustration of the 2D channel setup.

We consider a channel with length L and width $2a$, where $L \gg a$ is satisfied, as shown in Figure S11. The velocity field in the channel follows a Poiseuille profile:

$$u_{\mathrm{x}} \approx U\frac{3}{2}\left(1-\left(\frac{y}{a}\right)^2\right), \tag{14}$$

where U is the mean velocity magnitude. Initially, the channel is filled with an aqueous colloidal solution with the density $n_0 = 1$ and solute concentration c_0. At time $t = 0$, we inject an aqueous solution with $n_{\mathrm{in}} = 0$ and solute concentration $c_{\mathrm{in}} = c_1$ at the inlet at $x = 0$. The solute diffusivity is denoted by D_{s} and particle diffusivity by D_{p}. Here, we focus on the asymptotic regime for the solute transport when $t \gg \frac{a^2}{D_{\mathrm{s}}}$, namely the solute has reached the Taylor dispersion regime. Therefore, the solute concentration field can be expressed as [145]:

$$c(x,y,t) \approx c_0 + (c_1 - c_0)\left(X(x,t) + \frac{Pe}{8}\left(\frac{y}{a}\right)^2\left(2-\left(\frac{y}{a}\right)^2\right)a\frac{\partial X}{\partial x}\right), \tag{15}$$

where we define $Pe = \frac{Ua}{D_{\mathrm{s}}}$ and:

$$X(x,t) = \frac{1}{2}\left(1 - \mathrm{Erf}\left(\frac{x-Ut}{\sqrt{4\left(1+\frac{2}{105}Pe^2\right)D_{\mathrm{s}}t}}\right)\right). \tag{16}$$

Neglecting the (small) diffusiophoretic drift in the x-direction relative to the background flow, the effective velocity field experienced by the particles is

$$u_y = U\frac{\Gamma_{\mathrm{p}}}{2D_{\mathrm{s}}}\frac{y}{a}\left(1-\left(\frac{y}{a}\right)^2\right)\frac{a\,\partial X/\partial x}{X+\dfrac{1}{\beta-1}} = -U\frac{\Gamma_{\mathrm{p}}}{2D_{\mathrm{s}}}\frac{y}{a}\left(1-\left(\frac{y}{a}\right)^2\right)\frac{a}{\sqrt{4D_{\mathrm{eff}}t}}\exp\left[-\left(\frac{x-U_{\mathrm{m}}t}{\sqrt{4D_{\mathrm{eff}}t}}\right)^2\right], \tag{17}$$

with

$$u_x = U\frac{3}{2}\left(1-\left(\frac{y}{a}\right)^2\right), \qquad D_{\mathrm{eff}} = \left(1+\tfrac{2}{105}Pe^2\right)D_{\mathrm{s}}. \tag{18}$$

Equation (17) shows that the transverse diffusiophoretic drift decays *algebraically* and scales linearly with the background speed U, i.e., $u_y \sim Ua/\sqrt{D_{\mathrm{eff}}t}$. To estimate the net cross-stream displacement as a particle traverses the solute front, we note that solute gradients are mainly confined

within a diffusive width $w(t) \sim \sqrt{D_{\text{eff}} t}$. The approximate residence time of each particle within the front is therefore $\Delta t \sim w/U$. Consequently, the non-dimensional cross-stream coordinate displacement can be approximated as

$$\Delta y \sim u_y \Delta t \sim \frac{Ua}{\sqrt{D_{\text{eff}} t}} \cdot \frac{\sqrt{D_{\text{eff}} t}}{U} = \mathscr{O}(a), \tag{19}$$

indicating that the net displacement scales with the channel gap height.

Using this particle velocity field, we can derive particle trajectories under the assumption that particles are non-diffusive and $\frac{UL}{D_{\text{s}}^2} \gg 1$, from which the particle density field can be obtained. Moving the reference frame to that of the center of solute front and non-dimensionalizing the problem by defining $\bar{y} = \frac{y}{a}$, $\bar{x}_1 = \frac{x-Ut}{L}$, $\bar{t} = \frac{Ut}{L}$, $\bar{\Gamma}_{\text{p}} = \frac{\Gamma_{\text{p}}}{D_{\text{s}}}$, $\beta = \frac{c_1}{c_0}$, the particle density fields for the control, attractive and repulsive cases are obtained:

$$n_{\text{C}}(\bar{x}_1, \bar{t}) = \begin{cases} 0, \ \bar{x}_1 \in (-\infty, -\bar{t}] \\ n_0 \left(1 - \sqrt{\frac{1}{3}\left(1 - \frac{2\bar{x}_1}{\bar{t}}\right)}\right), \ \bar{x}_1 \in \left(-\bar{t}, \frac{1}{2}\bar{t}\right] \\ n_0, \ \bar{x}_1 \in \left(\frac{1}{2}\bar{t}, +\infty\right) \end{cases}, \tag{20}$$

$$n_{\text{A}}(\bar{x}_1, \bar{t}) = \begin{cases} 0, \ \bar{x}_1 \in (-\infty, -\bar{t}] \\ n_0 \beta^{-\bar{\Gamma}_{\text{p}}} \left(1 - \sqrt{\frac{1}{3}\left(1 - \frac{2\bar{x}_1}{\bar{t}}\right)}\right), \ \bar{x}_1 \in (-\bar{t}, 0] \\ n_0 (1 - \bar{y}_{\text{c}}), \ \bar{x}_1 \in \left(0, \frac{1}{2}\left(1 - 3(\bar{y}_{\text{c}})^2\right)\bar{t}\right] \\ n_0 \left(1 - \sqrt{\frac{1}{3}\left(1 - \frac{2\bar{x}_1}{\bar{t}}\right)}\right), \ \bar{x}_1 \in \left(\frac{1}{2}\left(1 - 3(\bar{y}_{\text{c}})^2\right)\bar{t}, \frac{1}{2}\bar{t}\right] \\ n_0, \ \bar{x}_1 \in \left(\frac{1}{2}\bar{t}, +\infty\right) \end{cases}, \tag{21}$$

$$n_{\text{R}}(\bar{x}_1, \bar{t}) = \begin{cases} 0, \ \bar{x}_1 \in (-\infty, -\bar{t}] \\ n_0 \beta^{-\bar{\Gamma}_{\text{p}}} \left(1 - \sqrt{\frac{1}{3}\left(1 - \frac{2\bar{x}_1}{\bar{t}}\right)}\right), \ \bar{x}_1 \in \left(-\bar{t}, \frac{1}{2}\left(1 - 3\bar{y}_{\text{d}}^2\right)\bar{t}\right] \\ n_0 \beta^{-\bar{\Gamma}_{\text{p}}} (1 - \bar{y}_{\text{d}}), \ \bar{x}_1 \in \left(\frac{1}{2}\left(1 - 3\bar{y}_{\text{d}}^2\right)\bar{t}, 0\right] \\ n_0 \left(1 - \sqrt{\frac{1}{3}\left(1 - \frac{2\bar{x}_1}{\bar{t}}\right)}\right), \ \bar{x}_1 \in \left(0, \frac{1}{2}\bar{t}\right] \\ n_0, \ \bar{x}_1 \in \left(\frac{1}{2}\bar{t}, +\infty\right) \end{cases}, \tag{22}$$

where $\bar{y}_{\text{c}} = -\frac{2}{\sqrt{3}} \cdot \cos\left(\frac{2\pi - \cos^{-1}\left(\beta^{-\bar{\Gamma}_{\text{p}}}\right)}{3}\right)$ and $\bar{y}_{\text{d}} = \frac{2}{\sqrt{3}} \cdot \cos\left(\frac{\pi - \cos^{-1}\left(\beta^{\bar{\Gamma}_{\text{p}}}\right)}{3}\right)$. Given the above particle density field, the equivalent dispersion coefficients can be derived by calculating the variance of $\frac{dn}{dt}(x,t)$ as a function of time t:

$$D_{\text{C}}^{\text{ND}} = \frac{U^2 t}{10}, \tag{23}$$

$$D_{\text{A}}^{\text{ND}} = \frac{U^2 t}{10} \cdot \beta^{-\bar{\Gamma}_{\text{p}}} \cdot \left(1 - \frac{\sqrt{3}}{6} - \frac{\sqrt{3}}{2}(\bar{y}_{\text{c}})^2 + \frac{2\sqrt{3}}{9}\frac{1}{1 - (\bar{y}_{\text{c}})^2}\right), \tag{24}$$

$$D_{\text{R}}^{\text{ND}} = \frac{U^2 t}{10} \cdot \left(\frac{\sqrt{3}}{6} + \frac{\sqrt{3}}{2}\bar{y}_{\text{d}}^2 - \bar{y}_{\text{d}}\beta^{-\bar{\Gamma}_{\text{p}}} + \beta^{-\bar{\Gamma}_{\text{p}}}\right), \tag{25}$$

where the contribution from the dispersion effect under the non-diffusive particle assumption in the control case, as shown in Eq. (23), aligns with previous work [145]. In the main text, we use the approximate form of these equations to describe the ratio of macroscopic dispersions in the attractive and repulsive cases over the control case. For the attractive case, when $\beta \gg 1$, $\bar{y}_{\mathrm{c}} \to 0$, so $D_{\mathrm{A}}^{\mathrm{ND}}/D_{\mathrm{C}}^{\mathrm{ND}} \approx (1 - \frac{\sqrt{3}}{6} + \frac{2\sqrt{3}}{9})\beta^{-\bar{\Gamma}_{\mathrm{p}}} \approx \beta^{-\bar{\Gamma}_{\mathrm{p}}}$. And for the repulsive case, when $\beta \ll 1$, $\bar{y}_{\mathrm{d}} \to 1 - \frac{1}{3\sqrt{3}}\beta^{\bar{\Gamma}_{\mathrm{p}}}$, so $D_{\mathrm{R}}^{\mathrm{ND}}/D_{\mathrm{C}}^{\mathrm{ND}} \approx (7\sqrt{3} - 3\beta^{\bar{\Gamma}_{\mathrm{p}}})/9$.

8. Influence of particle volume Fraction

The hydrodynamic disturbance velocity due to diffusiophoresis decays fast as $1/r^3$ away from each particle. This needs to be contrasted with the $1/r$ decay of particles driven by a body force, e.g., gravity. We, therefore, expect the hydrodynamic particle-particle interaction to be negligible in our experiments. Increasing the particle volume fraction beyond 10% could lead to deviations in the suspension viscosity, resulting in extra confinement felt by each particle and potentially inducing electrostatic interactions between the particles, which could then affect their spatial distribution and diffusiophoretic mobility.

We further conducted a few experiments using 10 times higher volume fraction of colloids, $\Phi = 3.8 \times 10^{-3}$. Since a higher volume fraction causes difficulties in detecting individual particles, we compare the normalized light intensity of the higher-volume-fraction case with the lower-volume-fraction case, $\Phi = 3.8 \times 10^{-4}$, which is the value used in the experimental dataset shown in the main manuscript.

Figure S12 shows that while changes in the volume fraction of colloids leads to quantitative differences, the qualitative influence of diffusiophoresis remains the same. It would certainly be interesting to systematically probe the role of volume fraction in future studies.

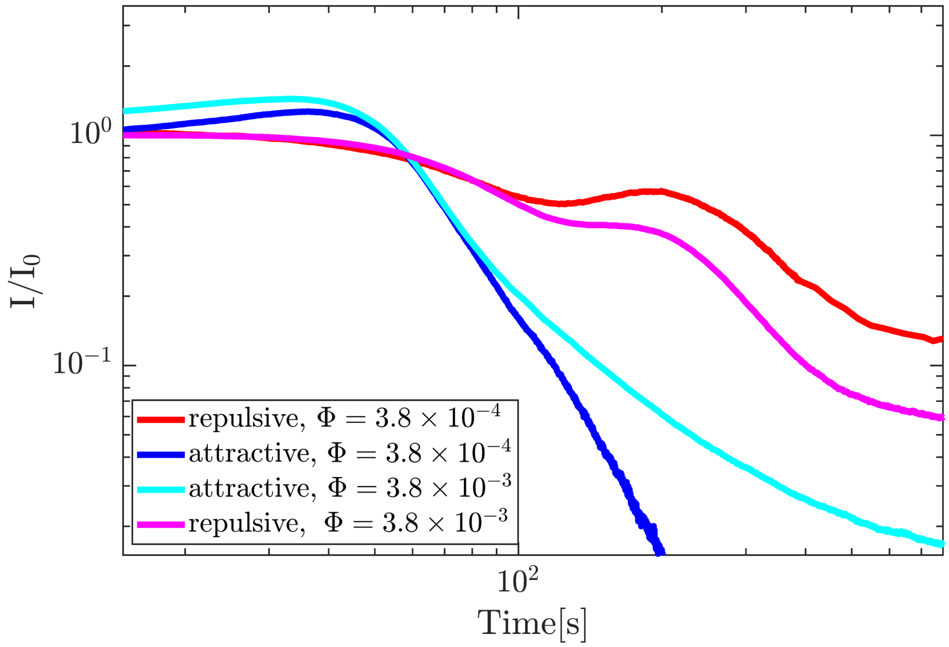

FIG. S12. Comparison of normalized particle light intensity as function of time for two different volume fraction, data shown in the main manuscripts refer to $\Phi = 3.8 \times 10^{-4}$.

REFERENCES

1. J. L. Anderson, Colloid transport by interfacial forces. *Annu. Rev. Fluid Mech.* **21**, 61–99 (1989).

2. D. Velegol, A. Garg, R. Guha, A. Kar, M. Kumar, Origins of concentration gradients for diffusiophoresis. *Soft Matter* **12**, 4686–4703 (2016).

3. S.Marbach, L. Bocquet, Osmosis, from molecular insights to large-scale applications. *Chem. Soc. Rev*. **48**, 3102 (2019).

4. S. Shin, Diffusiophoretic separation of colloids in microfluidic flows. *Phys. Fluids* **32**, 101302 (2020).

5. S. Shim, Diffusiophoresis, diffusioosmosis, and microfluidics: Surface-flow-driven phenomena in the presence of flow. *Chem. Rev.* **122**, 6986 (2022).

6. J. T. Ault, S. Shin, Physicochemical hydrodynamics of particle diffusiophoresis driven by chemical gradients. *Annu. Rev. Fluid Mech.* **57**, 227–255 (2025).

7. C. M. Gramling, C. F. Harvey, L. C.Meigs, Reactive transport in porous media: A comparison of model prediction with laboratory visualization. *Environ. Sci. Technol.* **36**, 2508–2514 (2002).

8. T. Borch, R. Kretzschmar, A. Kappler, P. V. Cappellen, M. Ginder-Vogel, A. Voegelin, K. Campbell, Biogeochemical redox processes and their impact on contaminant dynamics. *Environ. Sci. Technol.* **44**, 15–23 (2010).

9. M. Dentz, T. Le Borgne, A. Englert, B. Bijeljic, Mixing, spreading and reaction in heterogeneous media: A brief review. *J. Contam. Hydrol.* **120-121**, 1–17 (2011).

10. J. M. Matter, M. Stute, S. Ó. Snabjörnsdottir, E. H. Oelkers, S. R. Gislason, E. S. Aradottir, B. Sigfusson, I. Gunnarsson, H. Sigurdardottir, E. Gunnlaugsson, G. Axelsson, H. A. Alfredsson, D. Wolff-Boenisch, K. Mesfin, D. F. de la Reguera Taya, J. Hall, K. Dideriksen, W. S. Broecker, Rapid carbon mineralization for permanent disposal of anthropogenic carbon dioxide emissions. *Science* **352**, 1312–1314 (2016).

11. J. C. Stegen, J. K. Fredrickson, M. J. Wilkins, A. E. Konopka, W. C. Nelson, E. V. Arntzen, W. B. Chrisler, R. K. Chu, R. E. Danczak, S. J. Fansler, D. W. Kennedy, C. T. Resch, M. Tfaily, Groundwater–surface water mixing shifts ecological assembly processes and stimulates organic carbon turnover. *Nat. Commun.* **7**, 11237 (2016).

12. B. Berkowitz, I. Dror, S. K. Hansen, H. Scher, Measurements and models of reactive transport in geological media. *Rev. Geophys.* **54**, 930–986 (2016).

13. L. Li, K. Maher, A. Navarre-Sitchler, J. Druhan, C. Meile, C. Lawrence, J. Moore, J. Perdrial, P. Sullivan, A. Thompson, L. Jin, E. W. Bolton, S. L. Brantley, W. E. Dietrich, K. U. Mayer, C. I. Steefel, A. Valocchi, J. Zachara, B. Kocar, J. Mcintosh, B. M. Tutolo, M. Kumar, E. Sonnenthal, C. Bao, J. Beisman, Expanding the role of reactive transport models in critical zone processes. *Earth Sci. Rev*. **165**, 280–301 (2017).

14. B. Borer, R. Tecon, D. Or, Spatial organization of bacterial populations in response to oxygen and carbon counter-gradients in pore networks. *Nat. Commun.* **9**, 769 (2018).

15. M. Rolle, T. Le Borgne, Mixing and reactive fronts in the subsurface. *Rev. Mineral. Geochem.* **85**, 111–142 (2019).

16. J. Heyman, D. R. Lester, R. Turuban, Y. Méheust, T. Le Borgne, Stretching and folding sustain microscale chemical gradients in porous media. *Proc. Natl. Acad. Sci. U.S.A.* **117**, 13359–13365 (2020).

17. H. Sanquer, J. Heyman, K. Hanna, T. Le Borgne, Microscale chaotic mixing as a driver for chemical reactions in porous media. *Environ. Sci. Technol.* **58**, 8899–8908 (2024).

18. A. Velásquez-Parra, F. Marone, M. Griffa, J. Jiménez- Martínez, Chaotic transport of solutes in unsaturated porous media. *Environ. Sci. Technol.* **58**, 12643–12652 (2024).

19. C. A. Browne, S. S. Datta, Harnessing elastic instabilities for enhanced mixing and reaction kinetics in porous media. *Proc. Natl. Acad. Sci. U.S.A.* **121**, e2320962121 (2024).

20. A. Yang, B. E. McKenzie, B. Pavlat, E. S. Johnson, A. S. Khair, S. Garoff, R. D. Tilton, Diffusiophoretic transport of charged colloids in ionic surfactant gradients entirely below versus entirely above the critical micelle concentration. *Langmuir* **40**, 10143–10156 (2024).

21. S. Roman, F. Rembert, Inhibition of mineral dissolution by aggregation of colloidal particles driven by diffusiophoresis. *Phys. Rev. Fluids* **10**, L032501 (2025).

22. K. Müller, G. Magesan, N. Bolan, A critical review of the influence of effluent irrigation on the fate of pesticides in soil, agriculture. *Ecosyst. Environ.* **120**, 93–116 (2007).

23. Z. Steinmetz, C. Wollmann, M. Schaefer, C. Buchmann, J. David, J. Tröger, K. Muñoz, O. Frör, G. E. Schaumann, Plastic mulching in agriculture. Trading short-term agronomic benefits for long-term soil degradation? *Sci. Total Environ.* **550**, 690–705 (2016).

24. A. A. de Souza Machado, C. W. Lau, J. Till, W. Kloas, A. Lehmann, R. Becker, M. C. Rillig, Impacts of microplastics on the soil biophysical environment. *Environ. Sci. TEchnol.* **52**, 9656–9665 (2018).

25. M.MacLeod, H. P. H. Arp,M. B. Tekman, A. Jahnke, The global threat from plastic pollution. *Science* **373**, 61–65 (2021).

26. E. H. Lwanga, N. Beriot, F. Corradini, V. Silva, X. Yang, J. Baartman, M. Rezaei, L. van Schaik, M. Riksen, V. Geissen, Review of microplastic sources, transport pathways and correlations with other soil stressors: A journey from agricultural sites into the environment. *Chem. Biol. Technol. Agric.* **9**, 20 (2022).

27. D. Sheng, S. Jing, X. He, A.-M. Klein, H.-R. Köhler, T. C. Wanger, Plastic pollution in agricultural landscapes: An overlooked threat to pollination, biocontrol and food security. *Nat. Commun.* **15**, 8413 (2024).

28. A. A. Pahlavan, The soil plastisphere: The nexus of microplastics, bacteria, and biofilms. *InterPore J.* **1**, IPJ271124 (2024).

29. P. G. Tratnyek, R. L. Johnson, Nanotechnologies for environmental cleanup. *Nano Today* **1**, 44–48 (2006).

30. E. Lefevre, N. Bossa, M. R. Wiesner, C. K. Gunsch, A review of the environmental implications of in situ remediation by nanoscale zero valent iron (nZVI): Behavior, transport and impacts on microbial communities. *Sci. Total Environ.* **565**, 889 (2016).

31. T. Zhang, G. V. Lowry, N. L. Capiro, J. Chen, W. Chen, Y. Chen, D. D. Dionysiou, D. W. Elliott, S. Ghoshal, T. Hofmann, H. Hsu-Kim, J. Hughes, C. Jiang, G. Jiang, C. Jing, M. Kavanaugh, Q. Li, S. Liu, J. Ma, B. Pan, T. Phenrat, X. Qu, X. Quan, N. Saleh, P. J. Vikesland, Q. Wang, P. Westerhoff, M. S. Wong, T. Xia, B. Xing, B. Yan, L. Zhang, D. Zhou, P. J. J. Alvarez, In situ remediation of subsurface contamination: Opportunities and challenges for nanotechnology and advanced materials. *Environ. Sci. Nano* **6**, 1283–1302 (2019).

32. X. Huang, M. Auffan, M. J. Eckelman, M. Elimelech, J.-H. Kim, J. Rose, K. Zuo, Q. Li, P. J. J. Alvarez, Trends, risks and opportunities in environmental nanotechnology. *Nat. Rev. Earth Environ.* **5**, 572–587 (2024).

33. J. K. Jansson, N. Taş, The microbial ecology of permafrost. *Nat. Rev. Microbiol.* **12**, 414–425 (2014).

34. O. S. Pokrovsky, R. M. Manasypov, S. V. Loiko, L. S. Shirokova, Organic and organo-mineral colloids in discontinuous permafrost zone. *Geochim. Cosmochim. Acta* **188**, 118153 (2016).

35. M. A. Walvoord, B. L. Kurylyk, Hydrologic impacts of thawing permafrost—A review. *Vadose Zone J.* **15**, 1–20 (2016).

36. C. Hirst, E. Mauclet, A. Monhonval, E. Tihon, J. Ledman, E. A. G. Schuur, S. Opfergelt, Seasonal changes in hydrology and permafrost degradation control mineral element-bound DOC transport from permafrost soils to streams. *Global Biogeochem. Cycles* **36**, e2021GB007105 (2022).

37. Y. Jiao, Y. Zhang, X. Wang, I. Altshuler, F. Zhou, M. Fang, R. Rinnan, J. Chen, Z. Wang, Awakening: Potential release of dormant chemicals from thawing permafrost soils under climate change. *Environ. Sci. Technol.* **58**, 20336 (2024).

38. J. A. O'Donnell, M. P. Carey, J. C. Koch, C. Baughman, K. Hill, C. E. Zimmerman, P. F. Sullivan, R. Dial, T. Lyons, D. J. Cooper, B. A. Poulin,Metal mobilization from thawing permafrost to aquatic ecosystems is driving rusting of arctic streams. *Commun. Earth Environ.* **5**, 268 (2024).

39. E. K. Skierszkan, J. W. Dockrey, M. B. J. Lindsay, Metal mobilization from thawing permafrost is an emergent risk to water resources. *ACS ES&T Water* **5**, 20–32 (2025).

40. J. F. McCarthy, J. M. Zachara, Subsurface transport of contaminants. *Environ. Sci. Technol.* **23**, 496–502 (1989).

41. A. B. Kersting, D. W. Efurd, D. L. Finnegan, D. J. Rokop, D. K. Smith, J. L. Thompson, Migration of plutonium in ground water at the Nevada test site. *Nature* **397**, 56–59 (1999).

42. A. P. Novikov, S. N. Kalmykov, S. Utsunomiya, R. C. Ewing, F. Horreard, A. Merkulov, S. B. Clark, V. V. Tkachev, B. F. Myasoedov, Colloid transport of plutonium in the far-field of the mayak production association, Russia. *Science* **314**, 638–641 (2006).

43. F.-A.Weber, A. Voegelin, R. Kaegi, R. Kretzschmar, Contaminant mobilization by metallic copper and metal sulphide colloids in flooded soil. *Nat. Geosci.* **2**, 267–271 (2009).

44. S. A. Bradford, V. L. Morales, W. Zhang, R. Harvey, A. I. Packman, A. Mohanram, C. Welty, Transport and fate of microbial pathogens in agricultural settings. *Crit. Rev. Environ. Sci. Technol.* **43**, 775–893 (2013).

45. S. Wagner, A. Gondikas, E. Neubauer, T. Hofmann, F. vonderKammer, Spot the difference: Engineered and natural nanoparticles in the environment—Release, behavior, and fate. *Angew. Chem. Int. Ed. Engl.* **53**, 12398 (2014).

46. E. Spielman-Sun, K. Boye, D. Dwivedi, M. Engel, A. Thompson, N. Kumar, V. Noël, A critical look at colloid generation, stability, and transport in redox-dynamic environments: Challenges and perspectives. *ACS Earth Space Chem.* **8**, 630 (2024).

47. M. Elimelech, C. R. O'Melia, Kinetics of deposition of colloidal particles in porous media. *Environ. Sci. Technol.* **24**, 1528–1536 (1990).

48. D. Liu, P. R. Johnson, M. Elimelech, Colloid deposition dynamics in flow-through porous media: Role of electrolyte concentration. *Environ. Sci. Technol.* **29**, 2963 (1995).

49. J. N. Ryan, M. Elimelech, Colloid mobilization and transport in groundwater. *Colloids Surf. A. Physicochem. Eng. Asp.* **107**, 1–56 (1996).

50. P. R. Johnson, N. Sun, M. Elimelech, Colloid transport in geochemically heterogeneous porous media: Modeling and measurements. *Environ. Sci. Technol.* **30**, 3284 (1996).

51. S. B. Roy, D. A. Dzombak, Chemical factors influencing colloid-facilitated transport of contaminants in porous media. *Environ. Sci. Technol.* **31**, 656–664 (1997).

52. R. Kretzschmar, M. Borkovec, D. Grolimund, M. Elimelech, *Mobile Subsurface Colloids and Their Role in Contaminant Transport* (Academic Press, 1999), pp. 121–193.

53. S. A. Bradford, S. R. Yates, M. Bettahar, J. Simunek, Physical factors affecting the transport and fate of colloids in saturated porous media. *Water Resour. Res.* **38**, 63 (2002).

54. N. Tufenkji, M. Elimelech, Correlation equation for predicting single-collector efficiency in physicochemical filtration in saturated porous media. *Environ. Sci. Technol.* **38**, 529–536 (2004).

55. T. Kanti Sen, K. C. Khilar, Review on subsurface colloids and colloid-associated contaminant transport in saturated porous media. *Adv. Colloid Interface. Sci.* **119**, 71–96 (2006).

56. I. L. Molnar, W. P. Johnson, J. I. Gerhard, C. S. Willson, D. M. O'Carroll, Predicting colloid transport through saturated porous media: A critical review. *Water Resource. Res.* **51**, 6804 (2015).

57. F.Miele, P. de Anna, M. Dentz, Stochastic model for filtration by porous materials. *Phys. Rev. Fluids* **4**, 094101 (2019).

58. I. L. Molnar, E. Pensini, M. A. Asad, C. A. Mitchell, L. C. Nitsche, L. J. Pyrak-Nolte, G. L. Miño, M. M. Krol, Colloid transport in porous media: A review of classical mechanisms and emerging topics. *Transport Porous Med.* **130**, 129 (2019).

59. S. Aramideh, P. P. Vlachos, A. M. Ardekani, Nanoparticle dispersion in porous media in viscoelastic polymer solutions. *J. Nonnewton. Fluid Mech.* **268**, 75–80 (2019).

60. N. Bizmark, J. Schneider, R. D. Priestley, S. S. Datta,Multiscale dynamics of colloidal deposition and erosion in porous media. *Sci. Adv.* **6**, eabc2530 (2020).

61. D. Mangal, J. C. Palmer, J. C. Conrad, Nanoparticle dispersion in porous media: Effects of array geometry and flow orientation. *Phys. Rev. E* **104**, 015102 (2021).

62. D. Mangal, J. C. Conrad, J. C. Palmer, Nanoparticle dispersion in porous media: Effects of hydrodynamic interactions and dimensionality. *AIChE J.* **67**, e17147 (2021).

63. D. Mangal, J. C. Conrad, J. C. Palmer, Nanoparticle dispersion in porous media: Effects of attractive particle-media interactions. *Phys. Rev. E* **105**, 055102 (2022).

64. M. Kumar, J. S. Guasto, A. M. Ardekani, Transport of complex and active fluids in porous media. *J. Rheol.* **66**, 375–397 (2022).

65. D. Fan, E. Chapman, A. Khan, F. Iacoviello, G. Mikutis, *R. Pini*, A. Striolo, Anomalous transport of colloids in heterogeneous porous media: A multi-scale statistical theory. *J Colloid Interface Sci.* **617**, 94 (2022).

66. J. E. Patino,W. P. Johnson, V. L. Morales, Relating mechanistic fate with spatial positioning for colloid transport in surface heterogeneous porous media. *J Colloid Interface Sci.* **641**, 666–674 (2023).

67. T. Wu, Z. Yang, R. Hu, Y.-F. Chen, Three-dimensional visualization reveals pore-scale mechanisms of colloid transport and retention in two-phase flow. *Environ. Sci. Technol.* **57**, 1997–2005 (2023).

68. T. Wu, Y. Chen, Z. Yang, 3D pore-scale characterization of colloid aggregation and retention by confocal microscopy: Effects of fluid structure and ionic strength. *Sci. Total Environ.* **917**, 170349 (2024).

69. R. Storm, J. S. Marshall, Pore-scale modelling of particle transport in a porous bed. *J. Fluid. Mech.* **979**, A9 (2024).

70. W. K. Darko, D. Mangal, J. C. Conrad, J. C. Palmer, Particle dispersion through porous media with heterogeneous attractions. *Soft Matter* **20**, 837–847 (2024).

71. V. S. Doan, S. Chun, J. Feng, S. Shin, Confinement-dependent diffusiophoretic transport of nanoparticles in collagen hydrogels. *Nano Lett.* **21**, 7625–7630 (2021).

72. H. Tan, A. Banerjee, N. Shi, X. Tang, A. Abdel-Fattah, T. M. Squires, A two-step strategy for delivering particles to targets hidden within microfabricated porous media. *Sci. Adv.* **7**, eabh0638 (2021).

73. S. Sambamoorthy, H. C. W. Chu, Diffusiophoresis of a spherical particle in porous media. *Soft Matter* **19**, 1131–1143 (2023).

74. A. Somasundar, B. Qin, S. Shim, B. L. Bassler, H. A. Stone, Diffusiophoretic particle penetration into bacterial biofilms. *ACS Appl. Mater. Int.* **15**, 33263–33272 (2023).

75. S. Sambamoorthy, H. C. W. Chu, Diffusiophoresis in porous media saturated with a mixture of electrolytes. *Nanoscale Adv.* **7**, 2057–2067 (2025).

76. S. Shin, P. B.Warren, H. A. Stone, Cleaning by surfactant gradients: Particulate removal from porous materials and the significance of rinsing in laundry detergency. *Phys. Rev. Appl.* **9**, 034012 (2018).

77. S. W. Park, J. Lee, H. Yoon, S. Shin, Microfluidic investigation of salinity-induced oil recovery in porous media during chemical flooding. *Energy Fuels* **35**, 4885–4892 (2021).

78. M. Jotkar, P. de Anna, M. Dentz, L. Cueto-Felgueroso, The impact of diffusiophoresis on hydrodynamic dispersion and filtration in porous media. *J. Fluid Mech.* **991**, A8 (2024).

79. M. Jotkar, I. Ben-Noah, J. J. Hidalgo, M. Dentz, Diffusiophoresis of colloids in partially-saturated porous media. *Adv. Water Res.* **193**, 104828 (2024).

80. D. M. Walkama, N. Waisbord, J. S. Guasto, Disorder suppresses chaos in viscoelastic flows. *Phys. Rev. Lett.* **124**, 164501 (2020).

81. S. J. Haward, C. C. Hopkins, A. Q. Shen, Stagnation points control chaotic fluctuations in viscoelastic porous media flow. *Proc. Natl. Acad. Sci. U.S.A.* **118**, e2111651118 (2021).

82. B. Berkowitz, A. Cortis, M. Dentz, H. Scher, Modeling non-Fickian transport in geological formations as a continuous time random walk. *Rev. Geophys.* **44**, RG2003 (2006).

83. P. Gouze, Y. Melean, T. Le Borgne, M. Dentz, J. Carrera, Non-Fickian dispersion in porous media explained by heterogeneous microscale matrix diffusion. *Water Resour. Res.* **44**, W11416 (2008).

84. P. Gouze, T. Le Borgne, R. Leprovost, G. Lods, T. Poidras, P. Pezard, Non-Fickian dispersion in porous media: 1. Multiscale measurements using single-well injection withdrawal tracer tests. *Water Resour. Res.* **44**, W06426 (2008).

85. T. Le Borgne, P. Gouze, Non-Fickian dispersion in porous media: 2. Model validation from measurements at different scales. *Water Resour. Res.* **44**, W06427 (2008).

86. S. P. Neuman, D. M. Tartakovsky, Perspective on theories of non-Fickian transport in heterogeneous media. *Adv. Water Res.* **32**, 670–680 (2009).

87. B. Bijeljic, P. Mostaghimi, M. J. Blunt, Signature of non-Fickian solute transport in complex heterogeneous porous media. *Phys. Rev. Lett.* **107**, 204502 (2011).

88. P. de Anna, T. Le Borgne, M. Dentz, A. M. Tartakovsky, D. Bolster, P. Davy, Flow intermittency, dispersion, and correlated continuous time random walks in porous media. *Phys. Rev. Lett.* **110**, 184502 (2013).

89. A. Puyguiraud, P. Gouze, M. Dentz, Pore-scale mixing and the evolution of hydrodynamic dispersion in porous media. *Phys. Rev. Lett.* **126**, 164501 (2021).

90. A. D. Bordoloi, D. Scheidweiler,M. Dentz,M. Bouabdellaoui, M. Abbarchi, P. de Anna, Structure induced laminar vortices control anomalous dispersion in porous media. *Nat. Commun.* **13**, 3820 (2022).

91. A. Kar, T.-Y. Chiang, I. Ortiz Rivera, A. Sen, D. Velegol, Enhanced transport into and out of dead-end pores. *ACS Nano* **9**, 746–753 (2015).

92. S. Shin, E. Um, B. Sabass, J. T. Ault,M. Rahimi, P. B.Warren, H. A. Stone, Size-dependent control of colloid transport via solute gradients in dead-end channels. *Proc. Natl. Acad. Sci. U.S.A.* **113**, 257–261 (2016).

93. J. T. Ault, P. B. Warren, S. Shin, H. A. Stone, Diffusiophoresis in one-dimensional solute gradients. *Soft Matter* **13**, 9015–9023 (2017).

94. S. Battat, J. T. Ault, S. Shin, S. Khodaparast, H. A. Stone, Particle entrainment in dead-end pores by diffusiophoresis. *Soft Matter* **15**, 3879–3885 (2019).

95. N. Singh, G. T. Vladisavljević, F. M. C. Nadal, C. Cottin-Bizonne, C. Pirat, G. Bolognesi, Reversible trapping of colloids in microgrooved channels via diffusiophoresis under steady-state solute gradients. *Phys. Rev. Lett.* **125**, 248002 (2020).

96. J. L. Wilson, S. Shim, Y. E. Yu, A. Gupta, H. A. Stone, Diffusiophoresis in multivalent electrolytes. *Langmuir* **36**, 7014 (2020).

97. N. Shi, A. Abdel-Fattah, Droplet migration into dead-end channels at high salinity enhanced by micelle gradients of a zwitterionic surfactant. *Phys. Rev. Fluids* **6**, 053103 (2021).

98. B. M. Alessio, S. Shim, E. Mintah, A. Gupta, H. A. Stone, Diffusiophoresis and diffusioosmosis in tandem: Two dimensional particle motion in the presence of multiple electrolytes. *Phys. Rev. Fluids* **6**, 054201 (2021).

99. B. M. Alessio, S. Shim, A. Gupta, H. A. Stone, Diffusioosmosis-driven dispersion of colloids: A Taylor dispersion analysis with experimental validation. *J. Fliud Mech.* **942**, A23 (2022).

100. B. Akdeniz, J. A. Wood, R. G. H. Lammertink, Diffusiophoresis and diffusio-osmosis into a dead-end channel: Role of the concentration-dependence of zeta potential. *Langmuir* **39**, 2322 (2023).

101. R. E. Migacz, M. Castleberry, J. T. Ault, Enhanced diffusiophoresis in dead-end pores with time-dependent boundary solute concentration. *Phys. Rev. Fluids* **9**, 044203 (2024).

102. P. G. Saffman, A theory of dispersion in a porous medium. *J. Fliud Mech.* **6**, 321 (1959).

103. D. L. Koch, J. F. Brady, Dispersion in fixed beds. *J. Fliud Mech.* **154**, 399 (1985).

104. D. L. Koch, R. G. Cox, H. Brenner, J. F. Brady, The effect of order on dispersion in porous media. *J. Fliud Mech.* **200**, 173 (1989).

105. M. Dentz, M. Icardi, J. J. Hidalgo, Mechanisms of dispersion in a porous medium. *J. Fliud Mech.* **841**, 851 (2018).

106. M. Dentz, J. J. Hidalgo, D. Lester, Mixing in porous media: Concepts and approaches across scales. *Transport Porous Med.* **146**, 5 (2023).

107. R. Volk, C. Mauger, M. Bourgoin, C. Cottin-Bizonne, C. Ybert, F. Raynal, Chaotic mixing in effective compressible flows. *Phys. Rev. E* **90**, 013027 (2014).

108. J. Deseigne, C. Cottin-Bizonne, A. D. Stroock, L. Bocquet, C. Ybert, How a “pinch of salt” can tune chaotic mixing of colloidal suspensions. *Soft Matter* **10**, 4795 (2014).

109. L. Schmidt, I. Fouxon, D. Krug, M. van Reeuwijk, M. Holzner, Clustering of particles in turbulence due to phoresis. *Phys. Rev. E* **93**, 063110 (2016).

110. C. Mauger, R. Volk, N. Machicoane, M. Bourgoin, C. Cottin- Bizonne, C. Ybert, F. Raynal, Diffusiophoresis at the macroscale. *Phys. Rev. Fluids* **1**, 034001 (2016).

111. V. Shukla, R. Volk, M. Bourgoin, A. Pumir, Phoresis in turbulent flows. *N. J. Phys.* **19**, 123030 (2017).

112. F. Raynal, M. Bourgoin, C. Cottin-Bizonne, C. Ybert, R. Volk, Advection and diffusion in a chemically induced compressible flow. *J. Fluid Mech.* **847**, 228–243 (2018).

113. F. Raynal, R. Volk, Diffusiophoresis, batchelor scale and effective Péclet numbers. *J. Fluid Mech.* **876**, 818–829 (2019).

114. R. Volk, M. Bourgoin, C.-É. Bréhier, F. Raynal, Phoresis in cellular flows: From enhanced dispersion to blockage. *J. Fluid Mech.* **948**, A42 (2022).

115. P. Le-Clech, V. Chen, T. A. Fane, Fouling in membrane bioreactors used in wastewater treatment. *J. Membr. Sci.* **284**, 17–53 (2006).

116. A. Kar, R. Guha, N. Dani, D. Velegol, M. Kumar, Particle deposition on microporous membranes can be enhanced or reduced by salt gradients. *Langmuir* **30**, 793 (2014).

117. R. Guha, X. Shang, A. L. Zydney, D. Velegol, M. Kumar, Diffusiophoresis contributes significantly to colloidal fouling in low salinity reverse osmosis systems. *J. Membr. Sci.* **479**, 67–76 (2015).

118. H. Small, Hydrodynamic chromatography a technique for size analysis of colloidal particles. *J. Colloid Interface Sci.* **48**, 147–161 (1974).

119. D. C. Prieve, P. M. Hoysan, Role of colloidal forces in hydrodynamic chromatography. *J. Colloid Interface Sci.* **64**, 201–213 (1978).

120. M. T. Blom, E. Chmela, R. E. Oosterbroek, R. Tijssen, A. van den Berg, On-chip hydrodynamic chromatography separation and detection of nanoparticles and biomolecules. *Anal. Chem.* **75**, 6761–6768 (2003).

121. H. Zhang, Transport of microplastics in coastal seas. *Estuar. Coast. Shelf Sci.* **199**, 74–86 (2017).

122. X. Ouyang, C. M. Duarte, S.-G. Cheung, N. F.-Y. Tam, S. Cannicci, C. Martin, H. S. Lo, S. Y. Lee, Fate and effects of macro- and microplastics in coastal wetlands. *Environ. Sci. Technol.* **56**, 2386–2397 (2022).

123. T. Wang, S. Zhao, L. Zhu, J. C. McWilliams, L. Galgani, R. M. Amin, R. Nakajima,W. Jiang, M. Chen, Accumulation, transformation and transport of microplastics in estuarine fronts. *Nat. Rev. Earth Environ.* **3**, 795–805 (2022).

124. C.-H. Heldin, K. Rubin, K. Pietras, A. Östman, High interstitial fluid pressure—An obstacle in cancer therapy. *Nat. Rev. Cancer* **4**, 806–813 (2004).

125. H. Wiig, M. A. Swartz, Interstitial fluid and lymph formation and transport: Physiological regulation and roles in inflammation and cancer. *Physiol. Rev.* **92**, 1005 (2012).

126. M. J. Mitchell, M. M. Billingsley, R. M. Haley, M. E. Wechsler, N. A. Peppas, R. Langer, Engineering precision nanoparticles for drug delivery. *Nat. Rev. Drug Discov.* **20**, 101–124 (2021).

127. H. J. Keh, Diffusiophoresis of charged particles and diffusioosmosis of electrolyte solutions. *Curr. Opin. Colloid Interface Sci.* **24**, 13–22 (2016).

128. T.-Y. Chiang, D. Velegol, Multi-ion diffusiophoresis. *J. Colloid Interface Sci.* **424**, 120–123 (2014).

129. A. Gupta, B. Rallabandi, H. A. Stone, Diffusiophoretic and diffusioosmotic velocities for mixtures of valence-asymmetric electrolytes. *Phys. Rev. Fluids* **4**, 043702 (2019).

130. J. Heyman, Tractrac: A fast multi-object tracking algorithm for motion estimation. *Comput. Geosci.* **128**, 11 (2019).

131. D. C. Prieve, J. L. Anderson, J. P. Ebel, M. E. Lowell, Motion of a particle generated by chemical gradients. Part 2. Electrolytes. *J. Fluid Mech.* **148**, 247 (1984).

132. S. Shim, J. K. Nunes, G. Chen, H. A. Stone, Diffusiophoresis in the presence of a pH gradient. *Phys. Rev. Fluids* **7**, 110513 (2022).

133. B. J. Kirby, E. F. Hasselbrink Jr., Zeta potential of microfluidic substrates: 1. Theory, experimental techniques, and effects on separations. *Electrophoresis* **25**, 187–202 (2004).

134. B. J. Kirby, E. F. Hasselbrink Jr., Zeta potential of microfluidic substrates: 2. Data for polymers. *Electrophoresis* **25**, 203–213 (2004).

135. S. Shin, J. T. Ault, J. Feng, P. B. Warren, H. A. Stone, Low-cost zeta potentiometry using solute gradients. *Adv. Mater.* **29**, 1701516 (2017).

136. J. T. Ault, S. Shin, H. A. Stone, Characterization of surface–solute interactions by diffusioosmosis. *Soft Matter* **15**, 1582–1596 (2019).

137. I. Williams, S. Lee, A. Apriceno, R. P. Sear, G. Battaglia, Diffusioosmotic and convective flows induced by a nonelectrolyte concentration gradient. *Proc. Natl. Acad. Sci. U.S.A.* **117**, 25263–25271 (2020).

138. S. Lee, J. Lee, J. T. Ault, The role of variable zeta potential on diffusiophoretic and diffusioosmotic transport. *Colloids Surf. A. Physicochem. Eng. Asp.* **659**, 130775 (2023).

139. R. E. Migacz, G. Durey, J. T. Ault, Convection rolls and three-dimensional particle dynamics in merging solute streams. *Phys. Rev. Fluids* **8**, 114201 (2023).

140. A. Chakra, N. Singh, G. T. Vladisavljević, F. Nadal, C. Cottin- Bizonne, C. Pirat, G. Bolognesi, Continuous manipulation and characterization of colloidal beads and liposomes via diffusiophoresis in single- and double-junction microchannels. *ACS Nano* **17**, 14644–14657 (2023).

141. S. Zhang, H. C. W. Chu, Diffusioosmotic flow reversals due to ion–ion electrostatic correlations. *Nanoscale* **16**, 9367–9381 (2024).

142. S. Zhang, H. C. W. Chu, Competition between ion–ion electrostatic correlations and hydrodynamic slip radically changes diffusioosmosis. *Chem. Sci.* **15**, 18476 (2024).

143. H. Liu, A. A. Pahlavan, Diffusioosmotic reversal of colloidal focusing direction in a microfluidic T-junction. *Phys. Rev. Lett.* **134**, 098201 (2025).

144. B. Abecassis, C. Cottin-Bizonne, C. Ybert, A. Ajdari, L. Bocquet, Boosting migration of large particles by solute contrasts. *Nat. Mater.* **7**, 785–789 (2008).

145. R. Camassa, Z. Lin, R. M. McLaughlin, The exact evolution of the scalar variance in pipe and channel flow. *Commun. Math. Sci.* **8**, 601–626 (2010).

146. A. Gupta, S. Shim, H. A. Stone, Diffusiophoresis: From dilute to concentrated electrolytes. *Soft Matter* **16**, 6975–6984 (2020).

147. F. J. Meigel, T. Darwent, L. Bastin, L. Goehring, K. Alim, Dispersive transport dynamics in porous media emerge from local correlations. *Nat. Commun.* **13**, 5885 (2022).
